# Microbiome and tryptophan metabolomics analysis in adolescent depression: roles of the gut microbiota in the regulation of tryptophan-derived neurotransmitters and behaviors in human and mice

**DOI:** 10.1186/s40168-023-01589-9

**Published:** 2023-06-30

**Authors:** Manfei Zhou, Yichun Fan, Liuting Xu, Zheng Yu, Sizhe Wang, Huaisha Xu, Jiuping Zhang, Linwei Zhang, Wenwei Liu, Linlin Wu, Jing Yu, Honghong Yao, Jun Wang, Rong Gao

**Affiliations:** 1 Department of Hygienic Analysis and Detection, the Key Laboratory of Modern Toxicology of Ministry of Education, School of Public Health, Nanjing Medical University, 101 Longmian Avenue, Nanjing, 211166 China; 2 Department of Clinical Psychology, Nanjing Drum Tower Hospital, the Affiliated Hospital of Nanjing University Medical School, Zhongshan Road 321, Nanjing, 210008 China; 3 Nanjing Brain Hospital Affiliated to Nanjing Medical University, Nanjing, 210034 China; 4 Department of Toxicology, the Key Laboratory of Modern Toxicology of Ministry of Education, School of Public Health, Nanjing Medical University, 101 Longmian Avenue, Nanjing, 211166 China; 5 Wuxi Center for Disease Control and Prevention, Wuxi, 214023 China; 6 Department of Pharmacology, School of Medicine, Southeast University, Nanjing, 210096 China; 7 Center for Global Health, School of Public Health, Nanjing Medical University, Nanjing, 211166 China

**Keywords:** Gut microbiota, Adolescent depression, Tryptophan, Microbiota-gut-brain axis, Kynurenine, *Roseburia intestinalis*

## Abstract

**Background:**

Adolescent depression is becoming one of the major public health concerns, because of its increased prevalence and risk of significant functional impairment and suicidality. Clinical depression commonly emerges in adolescence; therefore, the prevention and intervention of depression at this stage is crucial. Recent evidence supports the importance of the gut microbiota (GM) in the modulation of multiple functions associated with depression through the gut-brain axis (GBA). However, the underlying mechanisms remain poorly understood. Therefore, in the current study, we aimed to screen the microbiota out from healthy and depressive adolescents, delineate the association of the targeted microbiota and the adolescent depression, address the salutary effects of the targeted microbiota on anti-depressive behaviors in mice involving the metabolism of the tryptophan (Trp)-derived neurotransmitters along the GBA.

**Results:**

Here, we found the gut microbiota from healthy adolescent volunteers, first diagnosis patients of adolescent depression, and sertraline interveners after first diagnosis displayed significant difference, the relative abundance of *Faecalibacterium*, *Roseburia*, *Collinsella*, *Blautia*, *Phascolarctobacterium*, *Lachnospiraceae-unclassified* decreased in adolescent depressive patients, while restored after sertraline treatment. Of note, the *Roseburia* abundance exhibited a high efficiency in predicting adolescent depression. Intriguingly, transplantation of the fecal microbiota from healthy adolescent volunteers to the chronic restraint stress (CRS)-induced adolescent depressed mice significantly ameliorated mouse depressive behaviors, in which the *Roseburia* exerted critical roles, since its effective colonization in the mouse colon resulted in remarkably increased 5-HT level and reciprocally decreased kynurenine (Kyn) toxic metabolites quinolinic acid (Quin) and 3-hydroxykynurenine (3-HK) levels in both the mouse brain and colon. The specific roles of the *Roseburia* were further validated by the target bacteria transplantation mouse model, *Roseburia intestinalis* (*Ri*.) was gavaged to mice and importantly, it dramatically ameliorated CRS-induced mouse depressive behaviors, increased 5-HT levels in the brain and colon via promoting tryptophan hydroxylase-2 (TPH2) or -1 (TPH1) expression. Reciprocally, *Ri.* markedly restrained the limit-step enzyme responsible for kynurenine (indoleamine2,3-dioxygenase 1, IDO1) and quinolinic acid (3-hydroxyanthranilic acid 3,4-dioxygenase, 3HAO) generation, thereby decreased Kyn and Quin levels. Additionally, *Ri*. administration exerted a pivotal role in the protection of CRS-induced synaptic loss, microglial activation, and astrocyte maintenance.

**Conclusions:**

This study is the first to delineate the beneficial effects of *Ri*. on adolescent depression by balancing Trp-derived neurotransmitter metabolism and improving synaptogenesis and glial maintenance, which may yield novel insights into the microbial markers and therapeutic strategies of GBA in adolescent depression.

Video Abstract

**Supplementary Information:**

The online version contains supplementary material available at 10.1186/s40168-023-01589-9.

## Background

Depression has affected more than 350 million people worldwide [1]. As a highly prevalent and recurrent disorder, half of onsets of depression occurs in adolescents [2]. Notably, women are much more prone to depression, as early onset of puberty in women results in chronic courses and recurrence of depression in adulthood [3]. Depression is ranked as the leading cause of disability and death among adolescents, and up to 20% of adolescents will experience major depressive disorder (MDD) before adulthood [4]. Despite being one of the greatest challenges to public health worldwide [5], depression remains poorly diagnosed and suboptimally treated due to its complex pathogenesis, individual differences among those afflicted, associated self-stigmas, and so on [6]. Therefore, it is of great value to explore the objective diagnostic indicators and potential mechanisms of the early stages of depression, especially in adolescence.

The microbiota-gut-brain (MGB) axis has been proposed to reveal the crosstalk between brain and intestinal flora, and this axis has emerged as a novel intervention target in depression [7]. An imbalance of neurotransmitters in the tryptophan (Trp) pathway, especially decrease of serotonin (5-hydroxytryptamine, 5-HT), a key metabolite of Trp, underlies the pathophysiology of depression. As a pivotal mediator of depression, numerous novel therapeutic strategies are based on the elevation of 5-HT levels to combat depression. Interestingly, over 90% of 5-HT in the body is produced in the gut, particularly in enterochromaffin cells (ECs); therefore, playing an indispensable role in the gut-brain axis. Noteworthily, increasing reports indicate that the influence of the Trp pathway exceeds the traditional focus on its metabolite 5-HT, and two other key branches of Trp, the kynurenine pathway (KP) and the indole pathway (IP), are involved in neuroendocrine activities [8, 9]. A growing body of research suggests that the KP is strongly correlated with mood disorders [10], for example, abnormal KP activation is found in both suicidal patients [11] and depression-like mice [12], with increased levels of toxic metabolites, such as kynurenine (Kyn) and quinolinic acid (Quin). Nonetheless, the full metabolic profile of the KP along the MGB axis in depression remains poorly understood.

The microbiome influences the gut-brain communication through endocrine, immune, and neuroactive pathways which are associated with depression. Fecal microbiota transplantation (FMT) allows researchers to decipher the association between the gut microbiota and depression by transplanting the “depression microbiota” into germ-free mice or microbiota-depleted rodents. This process can induce depression-like behaviors in recipient animals, demonstrating a critical role of the gut microbiome in depression onset [13]. However, most of the studies on the issue of depression-associated of microbiota to date have focused on the effects of the “health or depression microbiota” transplantation, whereas the mechanisms involving the adverse or beneficial effects of FMT on depression remain poorly understood. Based on the chronic restraint stress (CRS)-induced depressive mouse model (referred to hereafter as “CRS mouse model”), our recent study showed that microbiota at the genus level displayed significant differences among control, depressive, and citalopram-treated mice, which were highly correlated with the levels of Trp and its metabolites. In addition, CRS mice administered with *Parabacteroides distasonis.* displayed elevated concentration of 5-HT and higher ratio of 5-HT to Trp in the hippocampus, and concomitantly with the suppressed Kyn levels, thereby providing a novel insight into the potential effects of targeted microbiota intervention in depression [14].

In consideration of the critical roles of the neurotransmitters in depression and the fluctuated abundance of certain intestinal microbiota in depressive adolescents, we hypothesized that some specific composition(s) of enterogenous microbiome may associate with the depression-like behaviors. Thus, with the 16S sequencing assay, we screened the potential microbial biomarkers from healthy adolescent volunteers, first diagnosis patients of adolescent depression, and sertraline interveners. In addition, through single targeted bacteria implantation, the validation of certain microbial biomarker and the beneficial effects along the gut-brain axis (GBA) in adolescent depression was unraveled. Through the lens of gut microbiota and Trp-derived neurotransmitter metabolism, the present study revealed a previously unrecognized target microbiota-mediated improvement of Trp-5-HT and Trp-Kyn signaling metabolism and neural protection, thus providing a potentially unique therapeutic intervention in adolescent depression.

## Materials and methods

### Population samples

The depressive patients were recruited from Nanjing Brain Hospital affiliated to Nanjing Medical University (Jiangsu, China) from September 2020 to May 2021. Eligible female subjects (aged 11 to 17 years) were diagnosed by Diagnostic and Statistical Manual of Mental Disorders 5 (DSM-V) criteria and their symptom severity was quantified by Revised Child Anxiety and Depression Scale-25 (RCADS-25) [15]. In addition, depressive participants (*n* = 25) were excluded by parental history of bipolar disorder, schizophrenia or other psychiatric disorders according to DSM-V, meanwhile, subjects suffering from chronic systematic diseases, including diabetes, cardiovascular disease, thyroid disease, cancer etc., as well as substance abuse and infection records of bacteria, fungi or virus were also precluded. The healthy controls (HC, *n* = 10) were enrolled based on matching criteria of gender and other demographic information. The baseline demographic information of all participants was collected by the Child Mental Health Questionnaire. No candidates showed other major psychiatric comorbidities and all of them were drug-naive, without using antibiotics, anti-inflammatory medicine, prebiotics, or anti-depressants in the last 2 months. After samples (serum, urine, and feces) collection, the depressive subjects were further treated with sertraline for clinical normal course, and then evaluated by RCADS-25 for the second time. Subjects with lower scores, compared with the first time, were included in the recurrence group (DEP-sertraline treated). The protocols of clinical research were reviewed and approved by ECNMU (Approval No. 2020-KY198-01). All participants provided written informed consent prior to the study.

### Mice

Female C57/6 J mice (3 weeks old) were obtained from National Rodent Laboratory Animal Seed Center (Shanghai, China) and were maintained in the specific-pathogen-free level (SPF) environment with controlled room temperature (RT, 21–23 ℃), humidity (50–60%) and 12 h/12 h light/dark cycle. All mice had ad libitum access to dry food pellets and water and were left acclimatized for 1 week prior to any procedures. All the rodent experiments were approved by the Institutional Animal Care and Use Committee of Nanjing Medical University (Approval No. IACUC-2005051).

### Bacteria strain

*Roseburia intestinalis.* (*Ri.*, DSMZ 14610) strain was revitalized and cultured in YCFA broth (DSMZ Medium 1611) containing 5 mg Na-resazurin per liter (oxidation–reduction indicator). Sodium carbonate was added to the medium to adjust pH to 6.8 and the liquid medium was pre-reduced under anaerobic conditions for 24 h before culturing. The bacteria strain was then incubated in an anaerobic environment (gas atmosphere N_2_: CO_2_: H_2_ of 80:10:10) at 37 ℃ for 48–72 h. For the animal experiment, *Ri.* cells were collected by centrifugation (5000 rpm, 5 min, RT) and washed twice with sterile anaerobic PBS, finally re-suspended in 1 × PBS (OD_600_ = 0.2) and stored at 4 ℃ 1 day before mice gavage.

### Chronic restraint stress (CRS) procedure

According to our previous study [14], mice exposed to CRS were placed in the 50 mL restraint cylinders fitted closely to body size with holes which allowed free breathing for 14 consecutive days (3–4 h per day). The control (CTR) and CRS mice were then examined by the first-round behavioral tests in order to assess depression- and anxiety-like changes, following by random assignment with 4 sub-groups for the FMT or target bacteria intervention.

### Behavioral tests

Each of the experimental set (FMT set or target bacteria intervention set) contained two periods of behavioral tests. The first one was conducted after 2-week CRS modeling, while the other one was performed after microbiota transplantation or *Ri.* intervention. Mice were transferred to the testing room 3–4 h before all behavioral tests for adaption. In the spontaneous behavior experiments (open field test and elevated plus maze test), each mouse was gently placed in the cage frame or hands of the testers and was allowed free movement before formal tests. In the tail suspension test, open field test and elevated plus maze test, equipment was cleaned with 75% ethanol after every trial in order to minimize scents of the former test object. All the mice testing order was random.

### Sucrose preference test (SPT)

The SPT was performed based on the previous study [14]. Mice anhedonia was described by the consumption ratio of sucrose in this assay. After the last CRS, mice were housed individually and were acclimatized with two 50 mL drinking tubes for the first 24 h, both containing 1%(w/v) sucrose solution. In the next 24 h, one tube of the solution was replaced with drinking water and during the ensuing test period (24 h), mice were exposed to tubes with sucrose solution and water. The tube position was reversed every 12 h in order to avoid place preference. The liquid consumption was measured, and the sucrose preference was calculated as the fraction of the sucrose solution compared to the total drinking liquid amount.

### Open field test (OFT)

The OFT was implemented to evaluate mice spontaneous activity and anxiety-like behavior 1 day after SPT. Mice were placed in the center arena of a plastic open-field apparatus, consisting of one 50 × 50 cm base and four 50 × 30 cm walls, and were allowed to freely explore for 6 min. Time spent in different areas was recorded by ANY-maze software (Stoelting Co., USA).

### Elevated plus maze test (EPM)

Exploratory activity and anxiety-like behavior were measured in the elevated plus maze, which was 70 cm above the floor and composed of one central platform (5 × 5 cm), two open arms (30 × 5 cm) and two closed arms (30 × 5 × 20 cm). The closed ones were enclosed by non-transparent walls height in 20 cm. Mice were placed carefully in the center area and moved voluntarily for 6 min. Movement was recorded by ANY-maze software (Stoelting Co., USA).

### Tail suspension test (TST)

Each mouse was suspended with adhesive tape affixed 1–2 cm to the tip of the tail and inverted for 6 min, with its nose 20–25 cm away from the ground. Immobile state was considered as no body movement and passive hanging. The ratio of immobility was quantified by Tail Suspension-scan software (Geneandi Co., China) over the observation period to assess the behavioral despair.

### Forced swim test (FST)

Mice were individually placed in one plexiglass cylinder (diameter 30 cm, height 50 cm) filled with 30 cm deep water (23–25 ℃) for 6 min and videotaped in every session using Superfst software (Soft-maze software Co., China). Immobility was defined as the absence of all motion except for movements required to keep mouse’s head above the water.

### Treatments

According to Feng Z.’s protocol with some modifications [16], mice distributed into FMT section experienced antibiotics cocktail (ABX; vancomycin 50 mg/kg/day, neomycin 100 mg/kg/day, metronidazole 100 mg/kg/day, ampicillin 100 mg/kg/day, amphotericin-B 1 mg/kg/day) treatment for 1 week before microbiota inoculation. Ablation of endogenous gut flora was evaluated by aerobic/anaerobic plating on TSA plates according to Sun’s procedures [17]. Fecal samples harvested from the HC and DEP subjects were suspended with sterile phosphate buffer saline (PBS) at a dilution volume ratio of 1:10 (w/v, feces/PBS). Before centrifugation (1000 rpm, 5 min, 4 ℃), the solution was vortexed for further homogenate. Then the supernate was transferred and another centrifugation (8000 rpm, 5 min, 4 ℃) was applied for the bacterial precipitates. Mixtures were re-suspended by equivalent volume of sterile PBS for the transplantation protocol.

For the FMT part, every single mouse in transferring groups (CRS + HC-tr and CRS + DEP-tr) received 200 μL fecal suspension once every 2 days for 14 days by oral gavage, while the remaining groups (CTR and CRS) mice were administered by equivalent volume of sterile PBS simultaneously.

For the target bacteria intervention, mice were assigned to 4 groups (CTR + PBS, CTR + Ri, CRS + PBS and CRS + Ri) and were orally administered by sterile PBS or *Ri.* suspension (200 μL/per mouse) once a day for 14 consecutive days.

### Microbial DNA extraction and 16S sequencing

Human fecal samples (2–3 g) were collected in sterile stool cups and were stored at -80℃ until further processing. The microbial DNA was extracted by the E.Z.N.A. Stool DNA kit (Omega inc., USA) and the total DNA was eluted in 50 μL of elution buffer, stored at -80℃ until measurement in the PCR (LC-Bio Technology Co., Ltd., China). The V3-V4 region of the prokaryotic 16S rRNA gene was amplified with 341F (5′-CCTACGGGNGGCWGCAG-3) and 805R (5′-GACTACHVGGGT ATCTAATCC-3) [18], with specific barcodes tagged in the 5′ end, using PCR conditions (initial denaturation at 98 ℃ for 30 s, 32 cycles of denaturation at 98 ℃ for 10 s, annealing at 54 ℃ for 30 s and extension at 72 ℃ for 45 s). The products were confirmed with 2% agarose gel electrophoresis and then purified with AMPure XT beads (Beckman Coulter Genomics, USA), finally quantified using Qubit (Invitrogen, USA). Following amplicon library assessment, the libraries were sequenced on Illumina NovaSeq PE250 platform. Pair-end reads was assigned to samples based on their unique barcode and truncated by cutting off the barcode and primer sequence, then further merged into the longer tags according to their overlap parts, using FLASH (version 1.2.8). Quality scanning on the raw reads was performed under specific filtering conditions to obtain the high-quality clean tags by fqtrim (version 0.94). Chimeric sequences were filtered using Vsearch software (version 2.3.4). After de-replication by DADA2, amplicon sequence variants (ASV) feature table and feature sequences were obtained. Relative abundance was analyzed by QIIME2 process. Based on this, the diversity evaluations were conducted, including alpha diversity and beta diversity analysis. As to the species annotation, we used NT-16S database of SILVA (Release 138, https://www.arb-silva.de/documentation/release138/) for annotation, according to ASV feature sequences (The confidence threshold for annotation is 0.7). Other diagrams were implemented using R package (version 3.5.2).

### Western blotting

The prefrontal cortex (PFC; 30 mg) and clean colon tissues (2–3 cm) were collected in 2 mL screw cap microtubes containing 2 steel beads and 200–300 μL RIPA lysis buffer (25 mM Tris–HCl pH 7.6, 150 mM NaCl, 1% NP-40, 1% sodium deoxycholate, 0.1% SDS; Sigma-Aldrich, USA), supplemented with protease inhibitor cocktail (Sigma-Aldrich, USA). Homogenization (4800 rpm, 10 s, 6 cycles) was performed, immediately followed by tissue lysis on ice for 20 min, and then the lysed mixture was centrifuged in 12,000 rpm for 15 min at 4 ℃. The supernate was transferred to 1.5 mL EP tube and the protein concentration was determined by Pierce BCA Protein Assay Kit (Thermo Scientific Rockford, USA). Thirty micrograms of tissue lysates boiled with 5 × loading buffer (Beyotime, China) were loaded onto 10% or 12.5% SDS–polyacrylamide gradient gel (Epizyme, China) and subsequently transferred onto PVDF membranes (Millipore, USA), using liquid transfer system (Bio-Rad, USA). The membranes were then probed with diluted primary antibodies overnight at 4 ℃, after 2 h blocking with 3% bovine serum albumin (BSA) in Tris-buffered saline and Tween 20 (TBST; 10 mM Tris-base pH 7.6, 150 mM NaCl and 0.1% (v/v) Tween 20) at RT. Following washed with TBST, bands were incubated with secondary antibodies in blocking buffer (1:10,000) for 2 h at RT. Visualization was then implemented by Chemiluminescent HRP Substrate (Millipore, USA) and quantification was performed using Fiji software (Image J; NIH, USA).

### KP neurotransmitter quantification

The KP metabolites were determined using ultra-high performance liquid chromatography tandem mass spectrometry (UHPLC-MS/MS). Serum and supernate of homogenized tissue were thawed, mixed with isotopically labeled internal standard solutions and then transferred to new tubes after vortex and centrifugation (29,700 g, 10 min, 4 ℃). The cryogenic vacuum centrifugal enrichment and evaporation system (CentriVap Labconco) was applied for further derivatization. 50 μL dansyl chloride solution (2 mg/mL, diluted with acetone) and sodium bicarbonate buffer (pH = 9.0, 0.2 M) was mixed at 1:1 volume ratio, mixture was added to the dry matter and vortexed for 1 min. After water-bath heating, evaporation, re-dissolution, and centrifugation, 5 μL supernatant was injected.

### ELISA immunoassays

The kynurenine pathway metabolites were detected according to the manufacturer’s instructions. The ELISA kits including human 5-HT, DA, Trp, Kyn, Kyna, Quin, and NAD^+^ were used in the present work. Besides, parts of the KP metabolites of mice tissues were determined by ELISA kits. Data were normalized based on BCA determined protein concentrations.

### Immunofluorescence (IF) and confocal imaging

The PFC and distal colon tissues were employed in this assay. Perfused tissues were overnight immersed in 4% paraformaldehyde at 4 ℃, then embedded in paraffin, cut into 3–4 μm thick sections. After dewaxing and antigen repairing, the slices were blocked with 5% BSA buffer for 2 h at RT, followed by overnight incubation with primary antibodies diluted in the blocking solution (F-actin 1:200, Drebrin 1:50, PSD95 1:500, Syn1 1:200, GFAP 1:1000, Iba1 1:500, Occludin 1:400) at 4 ℃. Washed 3 times, the slices were further incubated with secondary antibodies for 2 h at RT, shielded from light. Then, DAPI solution was applied before slices sealed with glycerol. Samples were imaged by confocal microscopy using a Zeiss Laser Scanning Inverted Microscope LSM-710 and images were exported and analyzed by Black ZEN software (Carl Zeiss, Germany) and Fiji, respectively.

### Fluorescene in situ hybridization (FISH)

The paraffin-embedded colon tissues were subjected to deparaffinization, rehydration and permeabilization successively. The probe used in this study was designed according to Hold’s research [19] with some modifications, diluted with pre-warmed hybridization buffer (1:200, 88 ℃) and then balanced for 5 min at 37 ℃ before applying. Slices were blocked with 0.2 N HCl and Proteinase K (50 μg/mL) for 15 min, RT and 30 min, 37 ℃ respectively, further incubated with diluted probe solution in the dark and humid chamber at 37 ℃ overnight. Followed by washing with pre-warmed washing buffer at 37 ℃ for 15 min, slices were air dried and then added with 20 μL DAPI-antifade solution in dark environment and incubated for 5 min. Confocal images were acquired and analyzed, and the probe sequence was displayed in Table S2.

### Histological analysis

Formalin-fixed and paraffin-embedded colon tissues were sliced into 4-μm-thick sections and stained with hematoxylin and eosin (H&E) for histopathological examination. Images were scanned using Pannoramic scanner (3DHISTECH, Germany), and then the histological scores were calculated.

### qPCR analysis

Total RNA from mice colonic chunks (12 cm) was extracted using TRIZOL (Invitrogen, USA), while DNA from mice feces was isolated by Stool DNA extraction kit (TIANGEN, China). Chloroform and isopropanol were employed to precipitate the aqueous phase and RNA, or DNA concentration was examined by NanoDrop 2000 (Thermo Fisher, USA). cDNAs were generated from 500 ng total RNA with reverse transcription kit (Takara, Japan), followed by qPCR analysis using SYBR green kit (Takara, Japan). Primers sequences were listed in Table S2.

### Graphs and statistics

Data were analyzed using IBM SPSS 20.0 (Chicago, IL, USA) and GraphPad Prism 7 (San Diego, CA, USA), correlation images were created using R studio (Boston, MA, USA), and *p* ˂ 0.05 was considered statistically significant. Specific data significance values are reported in the figure legends, and most data are presented as mean ± SEM (except that the box chart shows the maximum and minimum values). Assumptions of normality and homogeneity of variance were checked using the Shapiro–Wilk and Levene tests, respectively. Significance between two independent groups that met the assumptions was determined using unpaired Student’s *t* tests, while those failed to meet the assumptions were examined using the Mann–Whitney *U* test. The chi-squared test was used for categorical data analysis. For multiple group comparisons, one-way ANOVA and two-way ANOVA followed by Tukey’s multiple comparison test were applied as appropriate. For the microbiome data analysis, due to its non-normal distribution, the Kruskal–Wallis test, along with Benjamini–Hochberg test were applied for multiple groups data analysis. Also, linear discriminant analysis effect size (LEfSe) analysis was used for comparing the differences in microflora composition of multiple groups. Receiver operating characteristic (ROC) curve was applied to depict the accuracy of targeted bacteria in distinguishing depressive adolescents and healthy controls. As to the type one error controlling, in the microbiota data analysis part, the Benjamini-Hochberg (original FDR method) was used for the multiple comparison among three groups. In the murine model data analysis part, the Tukey post hoc test was chosen due to its high testing efficiency in comparing equal or similar sample size data.

## Results

### Alterations of the intestinal microbiota in the unmedicated- and sertraline-treated-depressive adolescents

Healthy female controls (HC) and female unmedicated depressive (DEP) adolescents were enrolled in the present study, after the first diagnosis, the unmedicated patient samples were collected and the follow-up work was carried out for those patients who required sertraline treatment (DEP-sertraline-treated) until their depressive symptoms significantly improved, as evaluated by two attending psychiatrists or a clinician with a higher professional title. The HC adolescents were confirmed to be well matched with the depressive patients, as the majority of the demographic indicators were not significantly different between these two groups (Table S1).

**Fig. 1:**
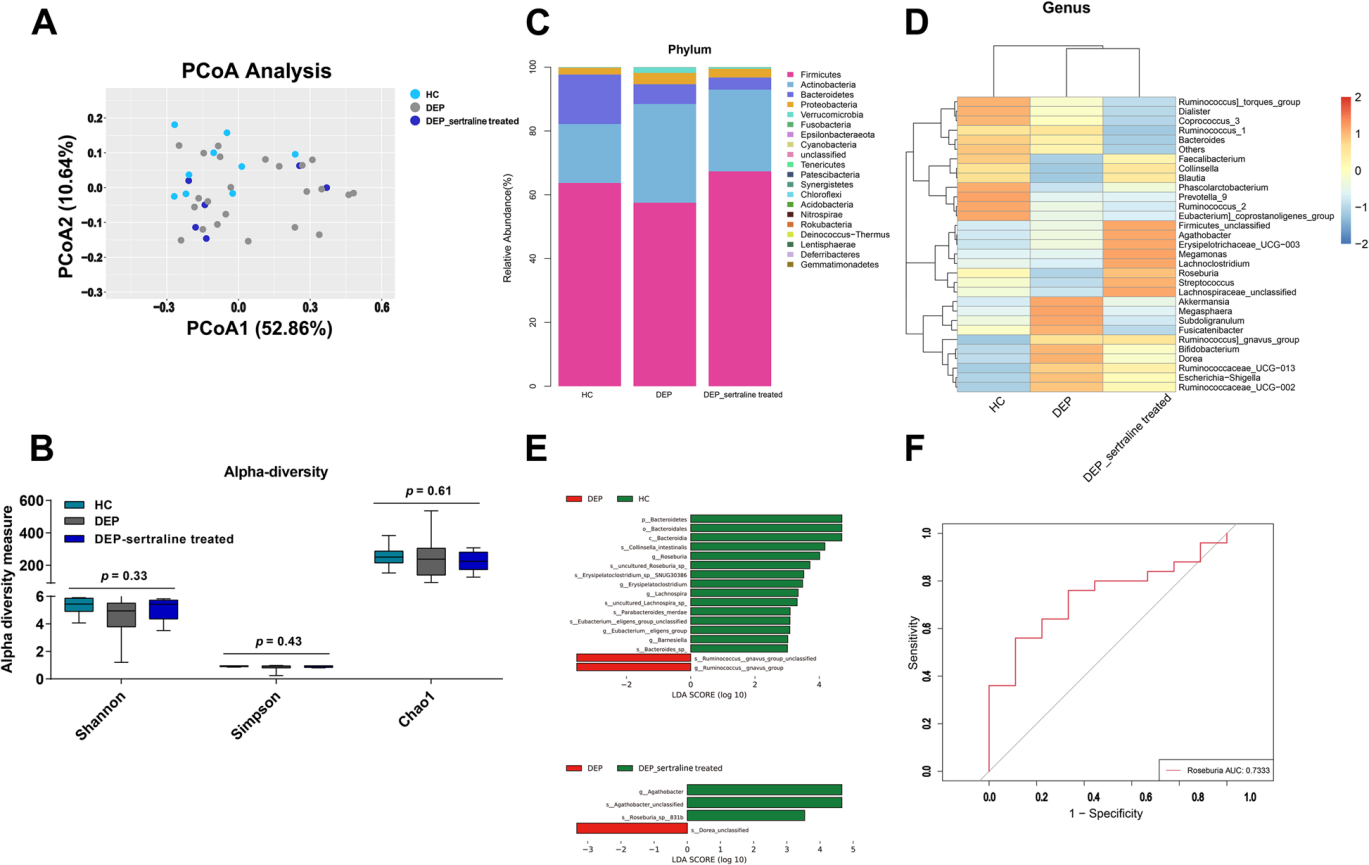
Alteration of GM in depressive adolescents. **A** Principal coordinate analysis of β diversity based on weighted UniFrac distances in the HC, DEP, and DEP-sertraline-treated groups. **B** Shannon, Simpson, and Chao1 indices of α diversity. **C** Stacked chart of GM composition at the phylum level. **D** Heatmap of the relative abundances of the top 30 microbiota genera. **E** Linear discriminant analysis effect size (LEfSe) analysis between group HC and DEP/ group DEP and DEP-sertraline-treated. **F** ROC curve of *Roseburia* in predicting adolescent depression according to binary logistic regression model between relative abundance of genus *Roseburia* and their groups (HC or DEP). Data were displayed as Minimum to Maximum in **B**. Significant differences among the three groups were determined via Kruskal-Wallis test

To ascertain the association between deviations in gut microbiome composition and depression, the 16S ribosomal DNA sequencing was performed. As shown in Figure S1A, 272 ASV features accounting for the total richness were common to all the groups, whereas 881 and 2329 ASVs accounted for HC and DEP groups, respectively, along with 369 ASVs accounted for DEP-sertraline-treated samples. According to principal coordinate analysis (PCoA), gut microbiota (GM) from the HC, DEP and DEP-sertraline-treated group exhibited difference (Fig. 1A), albeit no significant alteration in alpha diversity among the three groups, as indicated by the Shannon, Simpson and Chao1 indices (Fig. 1B). The taxonomy results showed that the phylum *Firmicutes* was enriched in both HC and DEP-sertraline-treated subjects, meanwhile, the relative abundance of *Actinobacteria*,* Proteobacteria*, and *Verrucomicrobia* phyla increased in adolescents with depression when compared with the HC group, and this was partially reversed after sertraline use (Fig. 1C). Notably, at the genus level, *Roseburia* was more abundant in HC while decreased in unmedicated depressive adolescents and restored in DEP-sertraline-treated groups (Fig. 1D and E, Figure S1B). The specific bacterial genus showed fair sensitivity and specificity, considering that the area under curve (AUC) reached 0.7333 in the ROC curve (cut-off point value = 1.10303, sensitivity = 0.89, specificity = 0.56, Fig. 1F), the *Roseburia* may serve as a promising prediction in depression development and treatment.

### Correlation analysis of the Trp-Kyn metabolic pathway, intestinal microbiota, and depressive symptoms in the unmedicated- and sertraline-treated-depressive adolescents

**Fig. 2:**
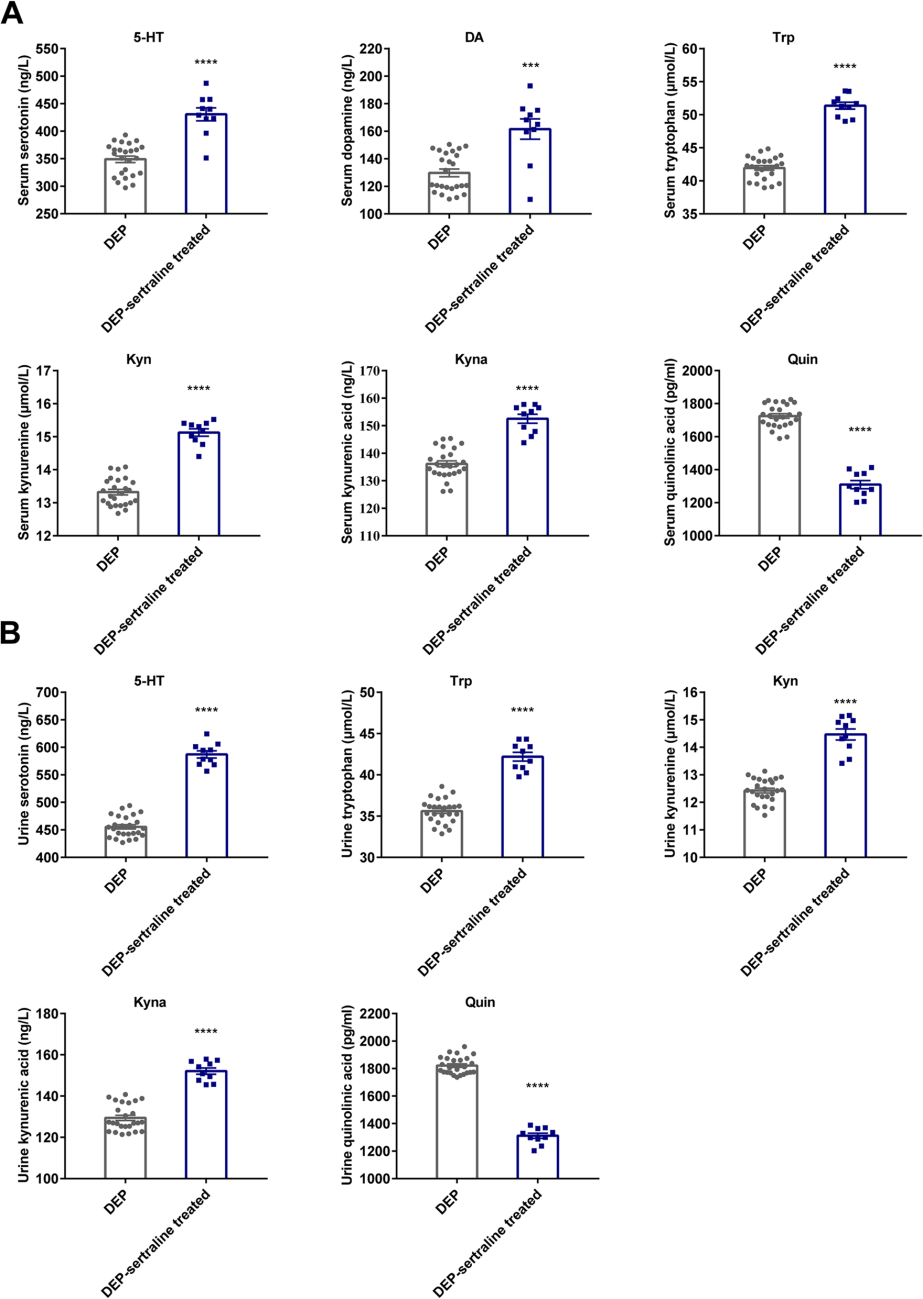
Alterations of Trp-Kyn pathway-derived metabolites in both serum and urine samples after antidepressant treatment. **A** Levels of dopamine and partial Trp-Kyn pathway metabolites in serum samples from depressive adolescents before and after sertraline treatment. **B** Levels of 5-HT and Trp-Kyn pathway metabolites in urine samples from depressive adolescents before and after sertraline treatment. Data were displayed as mean ± SEM. Significant differences before and after sertraline treatment were determined using Student’s *t* tests for serum 5-HT, Quin, and urine Trp, while Mann-Whitney *U* tests employed for other components. ^***^p < 0.001, ^****^p < 0.0001 *vs.* the DEP group

**Fig. 3:**
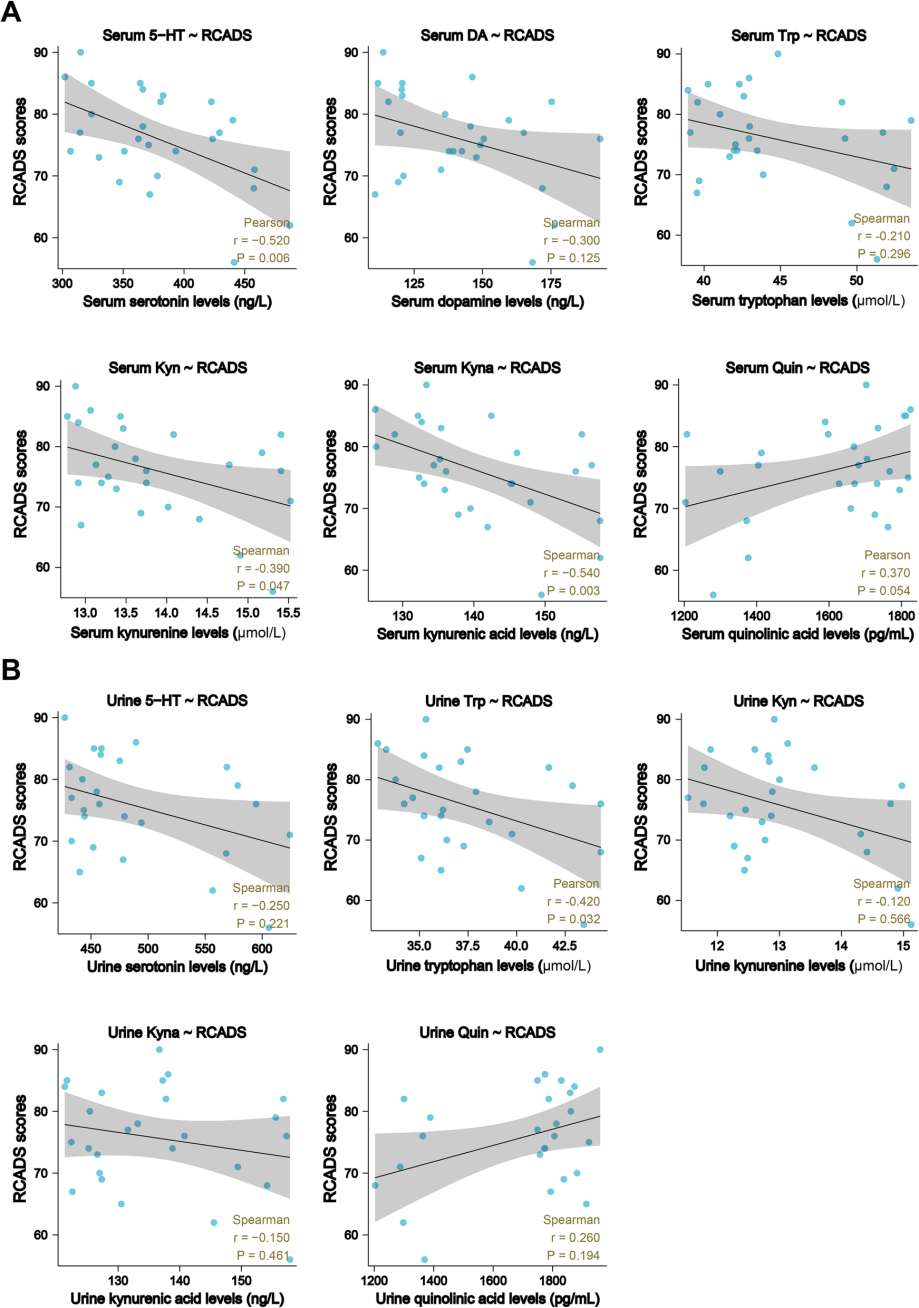
Correlations between adolescent depression scale scores and Trp-Kyn pathway metabolites. **A** Correlations between RCADS-25 scores and metabolite levels in serum. Correlations between RCADS-25 and 5-HT and Quin levels in serum were shown as Pearson’s *r* values. Correlations between RCADS-25 scores and DA, Trp, Kyn, and Kyna levels in serum were shown as Spearman’s *r* values. **B** Correlations between RCADS-25 scores and metabolite levels in urine. Correlation between RCADS-25 scores and Trp levels in urine is shown as Pearson’s *r* value. Correlations between RCADS-25 scores and 5-HT, Kyn, Kyna, and Quin levels in urine were shown as Spearman’s *r* values

Depression-like behavioral changes were highly associated with Trp-Kyn metabolism in the rodent model [14], thus, to fully unravel the alterations in Trp-derived metabolites (Trp, Kyn, kynurenic acid etc.) in adolescent depression, the neurotransmitters metabolized via the Trp pathway were examined. Compared with the unmedicated DEP group, the levels of Trp, 5-HT, and key KP metabolites, including Kyn and kynurenic acid (Kyna), were evidently increased in the serum and urine samples after the sertraline intervention, whereas Quin (serum and urine) and NAD^+^ (serum) levels were markedly suppressed (Fig. 2A, B, Figure S2B). To test whether the alterations in these neurotransmitters reflect the therapeutic effect of sertraline, a correlation analysis between the metabolite concentrations and RCADS-25 scores was conducted. In the serum, 5-HT, Kyn, and Kyna levels exhibited a pronounced negative association with self-rating scale scores (Fig. 3A), while the NAD^+^ level displayed a positive correlation (Figure S2C). In parallel with the results represented in the serum, a similar association between metabolite concentrations and RCADS-25 scores was observed in urine (Fig. 3B), suggesting that alterations in the targeted neurotransmitters specifically reflect the therapeutic effects of sertraline in adolescent depression.

### FMT from healthy adolescent volunteers ameliorates depression-like changes in CRS mice

**Fig. 4:**
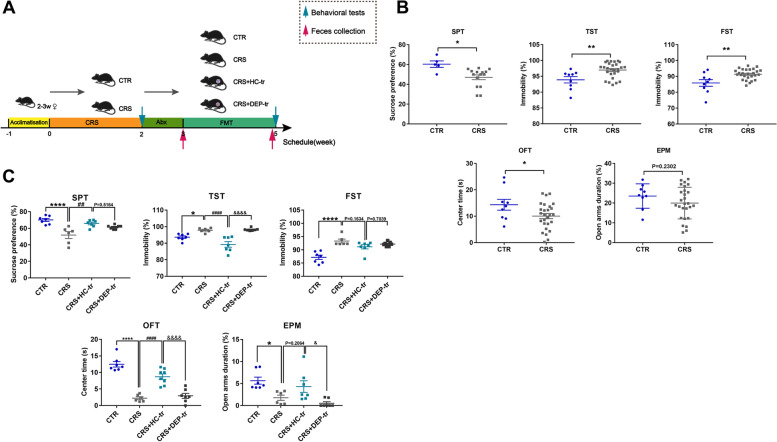
FMT from healthy volunteers ameliorated depression-like behaviors in mice. **A** Schematic illustration of the CRS and FMT procedures, with the respective groups labeled above the timeline. In brief, mice were randomly assigned to the CTR and CRS group after 1 week of acclimatization. The CRS modeling lasted for 2 weeks and was followed by the first behavioral tests (indicated by a blue arrow). Next, the mice in the FMT group (suffixed with -tr) were administered ABX, followed by feces collection (indicated by a red arrow) in order to verify the consumption of the native GM in the recipient mice. During FMT period (2 weeks), the mice in the CTR group were treated with 200 μL sterile PBS, while the CRS mice were further divided into 3 subgroups (CRS, CRS + HC-tr, and CRS + DEP-tr), with the CRS + HC-tr and CRS + DEP-tr mice receiving 200 μL of the feces microbiota suspension from the HC and DEP adolescents, respectively. At the end of the FMT, all mice were examined during the second round of behavioral tests (indicated by a blue arrow). **B** The first round of behavioral tests with measures including sucrose consumption (%) in the SPT, immobility (%) in the TST and FST, center duration (s) in the OFT, and open arms duration (%) in the EPM test. **C** The second round of behavioral tests in the four subgroups mice. Data were displayed as mean ± SEM. Significant differences were determined via Student’s *t* test or one-way ANOVA and Tukey’s multiple comparison procedure. ^*^p < 0.05, ^**^p < 0.01, ^****^p < 0.0001 *vs.* the CTR group; ^##^*p* < 0.01, ^####^*p* < 0.0001 *vs.* the CRS group; ^&^*p* < 0.05, ^&&&&^*p* < 0.0001 *vs.* the CRS + HC-tr group

To unravel the relationship among GM, KP metabolites, and adolescent depression, a human-to-mice FMT model was established (Fig. 4A) to determine whether an intestinal flora alteration can affect depressive behaviors by manipulating a host’s metabolism. In this study, 3-week-old female mice were used, corresponding to the higher prevalence of female adolescents in epidemiological studies [20]. The behavioral tests showed that the adolescent CRS mice exhibited obvious depression- and anxiety-like behaviors compared with the control (CTR) mice, with less consumption of sucrose solution in SPT and increased immobility states in both the TST and FST experiments. Meanwhile, the OFT results showed that CRS mice obviously spent less time in the center arena, and the EPM test data exhibited that they spent less time in the open arms, although the difference was not pronounced (Fig. 4B). These results demonstrated that depression-like behaviors in mice were induced by CRS. To assess whether the healthy human-to-mice FMT benefits CRS-induced depression, a gut microbiota depletion mouse model was constructed with antibiotic cocktail (ABX). Following 1-week ABX gavage and 2-week FMT, the depression-like behaviors in the SPT and TST of the adolescent CRS mice were markedly ameliorated after FMT from HC adolescents, and these changes were concomitant with relieved anxiety-like alterations in the OFT (Fig. 4C). In parallel, the gut microbiota transplanted from depressive adolescents showed no exacerbated effects on CRS-induced mice. Taken together, these results implied that some microbiota in the healthy adolescent feces may exert beneficial effects on CRS-induced depressive mice.

### FMT from healthy adolescent volunteers ameliorates CRS-induced neurotransmitter perturbation through the rate-limiting enzymes in mice

**Fig. 5:**
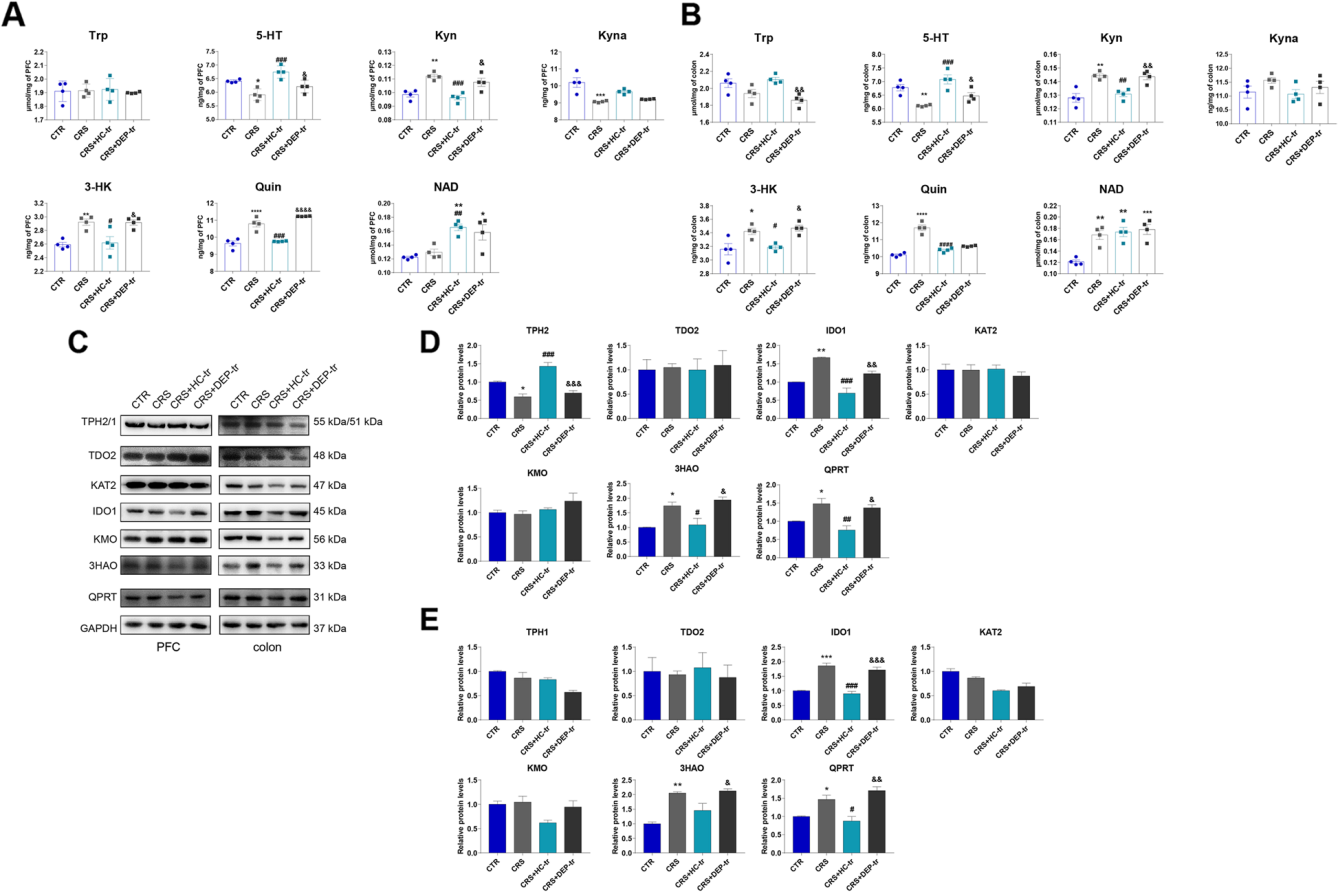
FMT from healthy volunteers ameliorated the neurotransmitter perturbation by the Trp-Kyn pathway by affecting the rate-limiting enzymes in both the brain and colon. **A** Levels of Trp-Kyn metabolic pathway-derived neurotransmitters in the mice PFCs. **B** Levels of Trp-Kyn metabolic pathway-derived neurotransmitters in the mice colons. **C** Protein expression of the rate-limiting enzymes of the Trp-Kyn Pathway in the mice PFCs and colons. **D** Statistical plots of the mice PFCs. **E** Statistical plots of the mice colons. Data were displayed as mean ± SEM. Significant differences were determined via one-way ANOVA and Tukey’s multiple comparison procedure. ^*^p < 0.05, ^**^p < 0.01 *vs.* the CTR group; ^#^*p* < 0.05, ^##^*p* < 0.01, ^###^*p* < 0.001 *vs.* the CRS group; ^&^*p* < 0.05, ^&&^*p* < 0.01, ^&&&^*p* < 0.001 *vs.* the CRS + HC-tr group

Neurotransmitter metabolism is strongly linked to depression- and anxiety-like behaviors, and Trp is a precursor of 5-HT and neuroactive KP metabolites [21]. Based on the depressive phenotype observed in the adolescent FMT rodent model aforementioned, the Trp-derived metabolic neurotransmitters were examined. As shown in Fig. 5A, B, a pronounced decrease in 5-HT in the PFC and colon was observed in the CRS mice, a process that was appreciably rescued following HC-GM transplantation. Reciprocally, Kyn levels were significantly elevated in the brains and guts of both CRS and CRS + DEP-tr mice, while microbiota from HC adolescents strikingly suppressed KP activation, as the levels of Kyn and its downstream neurotoxic metabolites, namely 3-HK and Quin, dramatically decreased (Fig. 5A, B). Similar results were also observed in the mouse serum, where the decreased levels of 5-HT were accompanied by increased levels of toxic KP products (3-HK, 3-HAA, and Quin), which were partially reversed by the HC microbiome transfer (Figure S3B). Moreover, the 5-HT pathway metabolites, including N-acetylserotonin (NAS) and melatonin (MLT), in the PFC were downregulated in CRS and CRS + DEP-tr mice, and these neurotransmitters were enhanced in the HC-GM treatment group (Figure S3A). Interestingly, the NAD^+^ levels increased in the CRS-treated mice, whereas the Trp levels showed no pronounced changes in the PFC, serum, and colon samples among the four groups (Fig. 5A, B and Figure S3B). Moreover, evidence for KP activation was also indicated in the CRS mice serum, as the metabolites of the KP, including Kyn, Kyna, 3-HK, 3-HAA, Quin, and NAD^+^ were significantly increased or displayed an increased tendency; synchronously, the 5-HT level in the serum was obviously reduced in the CRS-treated group (Figure S3B). To decipher the mechanisms of the neurotransmitter alterations mediated by CRS, the rate-limiting enzymes responsible for maintaining the balance of these neurotransmitters were dissected. As shown in Fig. 5C, D, the quantitative immunoblot assay displayed an evident decrease in tryptophan hydroxylase-2 (TPH2), an enzyme that plays a key role in 5-HT generation in the brain. Meanwhile, the levels of KP rate-limiting enzymes indole-amine oxygenase-1 (IDO1), 3-Hydroxyanthranilic acid 3, 4-dioxygenase (3HAO), and quinolinate phosphoribosyl transferase (QPRT) were markedly increased in both the PFC and colon samples from CRS and CRS + DEP-tr mice, and these effects could be rescued by HC-GM transplantation (Fig. 5C–E). Taken together, these results implied that some microbiota in the healthy adolescent feces modulate the rate-limiting enzymes, thereby, regulate the neurotransmitter metabolism both in the brain and colon.

### FMT from healthy adolescent volunteers is beneficial to synaptic plasticity and glial homeostasis in adolescent depressive mice

**Fig. 6:**
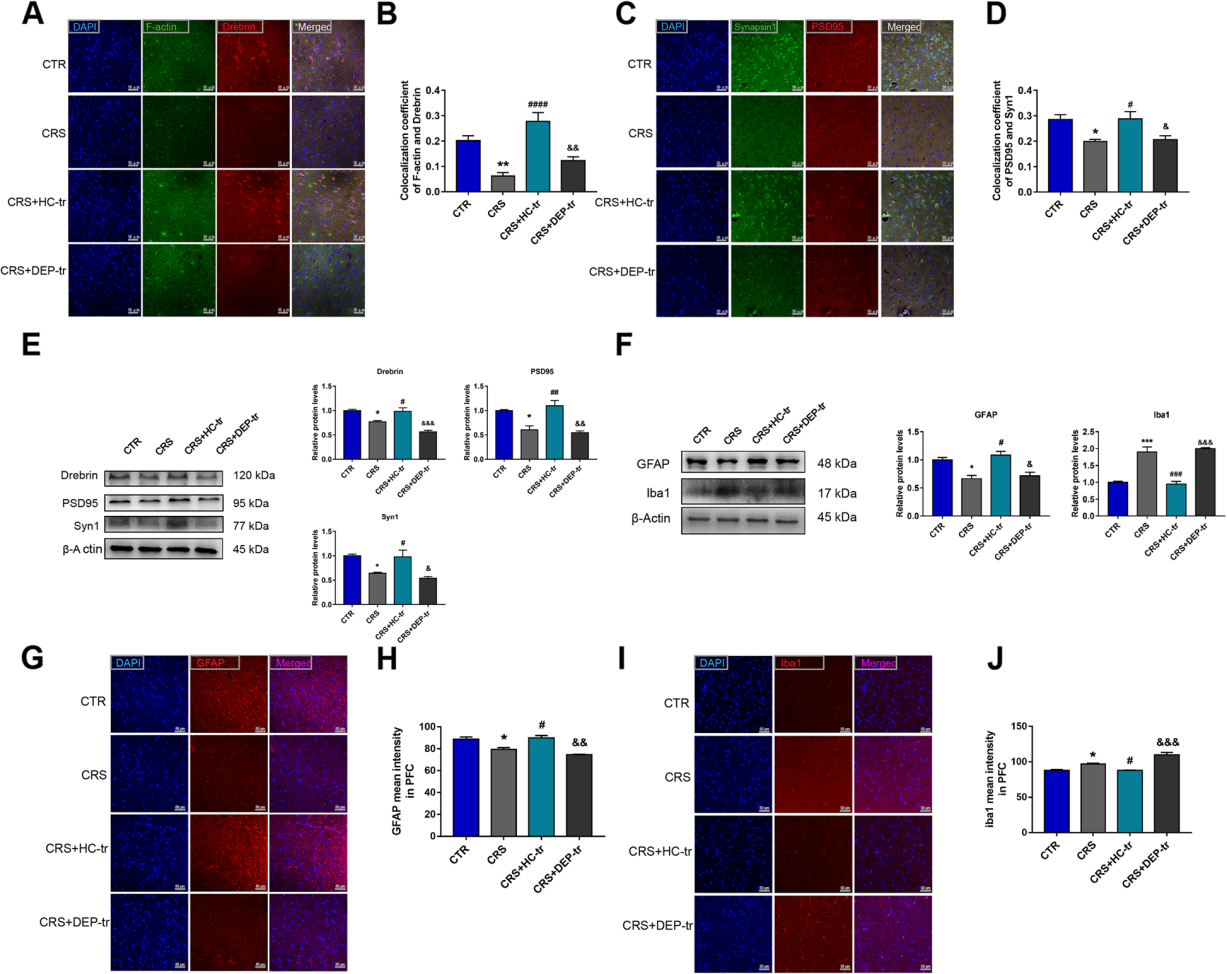
FMT from healthy volunteers improved the synaptic plasticity and glial activities induced by CRS in mouse brains. **A**, **B** Colocalization of F-actin and Drebrin along with the respective coefficient analysis. **C**, **D** Colocalization of Synapsin1 and PSD95 along with the respective coefficient analysis. **E** Protein expression of Drebrin, PSD95, and Syn1 along with their statistical graphs. **F** Protein expression of GFAP and Iba1 along with their statistical graphs. **G**, **H** MFI of GFAP and their statistical graphs. **I**, **J** MFI of Iba1 and their statistical graphs. Scale bar 50 μm. Data were displayed as mean ± SEM. Significant differences were determined via one-way ANOVA and Tukey’s multiple comparison procedure. ^*^p < 0.05, ^**^p < 0.01, ^***^p < 0.001 *vs.* the CTR group; ^#^*p* < 0.05, ^##^*p* < 0.01, ^###^*p* < 0.001, ^####^*p* < 0.0001 *vs.* the CRS group; ^&^*p* < 0.05, ^&&^*p* < 0.01, ^&&&^*p* < 0.001 *vs.* the CRS + HC-tr group

The KP downstream metabolites, such as Kyna and Quin, play essential roles in synaptic plasticity, which are involved in the progression of adolescent depression [22]. Thus, the density of dendritic spines and synapses in the PFC was examined by double labeling with F-actin/Drebrin and Synapsin1 (Syn1)/Postsynaptic Density Protein95 (PSD95), respectively. As depicted in Fig. 6A–D, the HC fecal transplantation in adolescent CRS mice displayed more colocalization of F-actin and Drebrin, coupled with increased co-expression of Syn1 and PSD95 in the PFC. In accordance with the immunofluorescent results, CRS treatment remarkably decreased the protein levels of Drebrin, Syn1, and PSD95, whereas FMT from healthy adolescents substantially reversed these effects (Fig. 6E), suggesting that the normobiotic GM functions as a manipulator of synapse remodeling.

Microglial cells and astrocytes are vital sources of KP products in the brain [23]. Activated microglial cells are not only responsible for immune homeostasis in the brain, but also produce neurotoxic KP metabolites, such as Quin [24], under conditions of inflammation, which likely aggravates depressive symptoms in adolescents [25]. Interestingly, the activity undertaken in astrocytes, to some extent, exhibits neuroprotective effects by assuming the generation of Kyna, an effective neuroprotective agent in the case of excitotoxicity [26]. In this study, we evaluated the activities of both microglia and astrocytes. As shown in Fig. 6F–H, the protein level and mean fluorescence intensity (MFI) of GFAP increased in CRS + HC-tr group, compared to in adolescent CRS mice, indicating enhanced activation of astrocytes by HC-GM transplantation; meanwhile, as expected, Iba1-marked microglial activation was significantly ameliorated following normobiotic GM transplantation (Fig. 6F, I, J). These results, taken together, suggesting that FMT from healthy adolescent volunteers retards CRS induced depression-like changes may partially through ameliorating synaptic loss, microglial activation and improving astrocyte activity.

### FMT from healthy adolescent volunteers restores CRS-induced colonic impairment

**Fig. 7:**
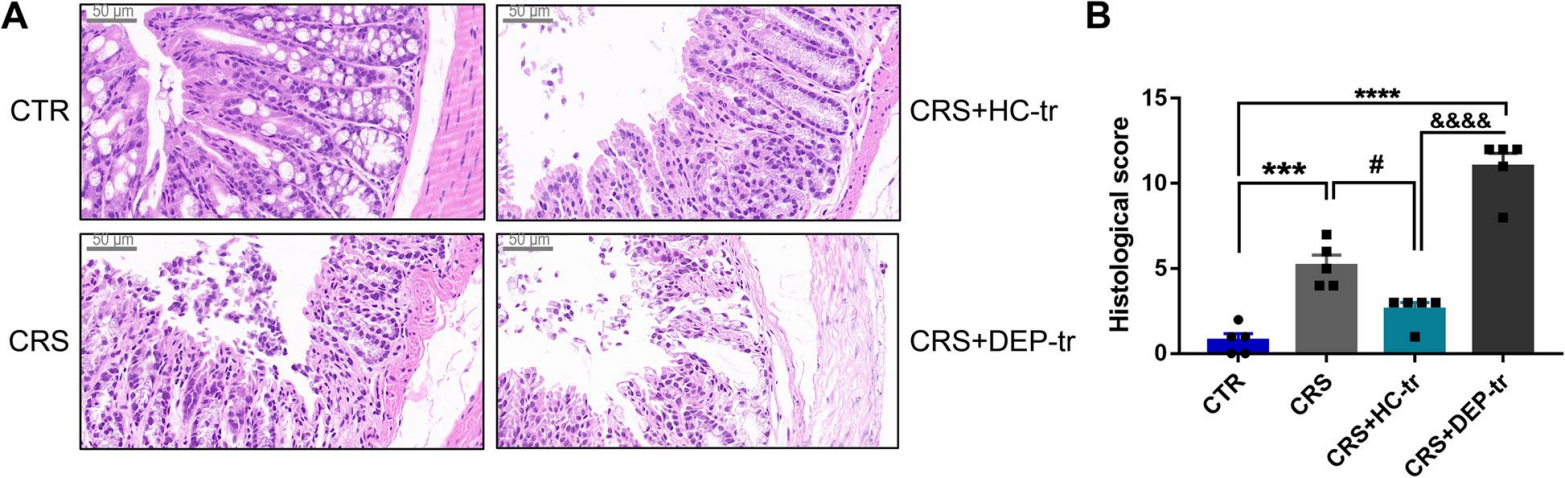
FMT from healthy volunteers ameliorated colonic epithelium injuries induced by CRS. **A** H&E staining of the colons of differently treated mice. Scale bar 50 μm. **B** Histological score analysis of the differently treated mice. Data were displayed as mean ± SEM. Significant differences were determined via one-way ANOVA and Tukey’s multiple comparison procedure. ^***^p < 0.001, ^****^p < 0.0001 *vs.* the CTR group; ^#^*p* < 0.05 *vs.* the CRS group; ^&&&&^*p* < 0.0001 *vs.* the CRS + HC-tr group

Perturbation of microbial homeostasis in the intestine is vulnerable to gut barrier damage and may further develop into “leaky gut” [27]. Lipopolysaccharide (LPS), along with other pro-inflammatory factors and toxic products, can easily translocate into the peripheral circulation and cause adverse effects on the CNS, such as synaptic injury and glial activation [28]. Since the pronounced increase of the toxic metabolites such as Kyn and Quin induced by CRS in the mouse colon, then the colonic structure integrity of the mice in this study was evaluated using their histological scores (Fig. 7A, B). CRS was found to cause evident epithelial structure deficiencies, manifesting as crypt distortion and goblet cell atrophy, which were remarkably recovered by HC-GM transplantation. Notably, DEP microbiota transplantation caused even worse barrier disruption, as the histological score in CRS + DEP-tr mice was higher than that in the CRS group (Fig. 7B). Collectively, FMT from healthy adolescent volunteers protects against CRS induced gut barrier damage, which may alleviate synapse injury and glial activation along the GBA.

### *Roseburia Intestinalis.* (*Ri.*) colonization ameliorates depressive behaviors in adolescent mice

**Fig. 8:**
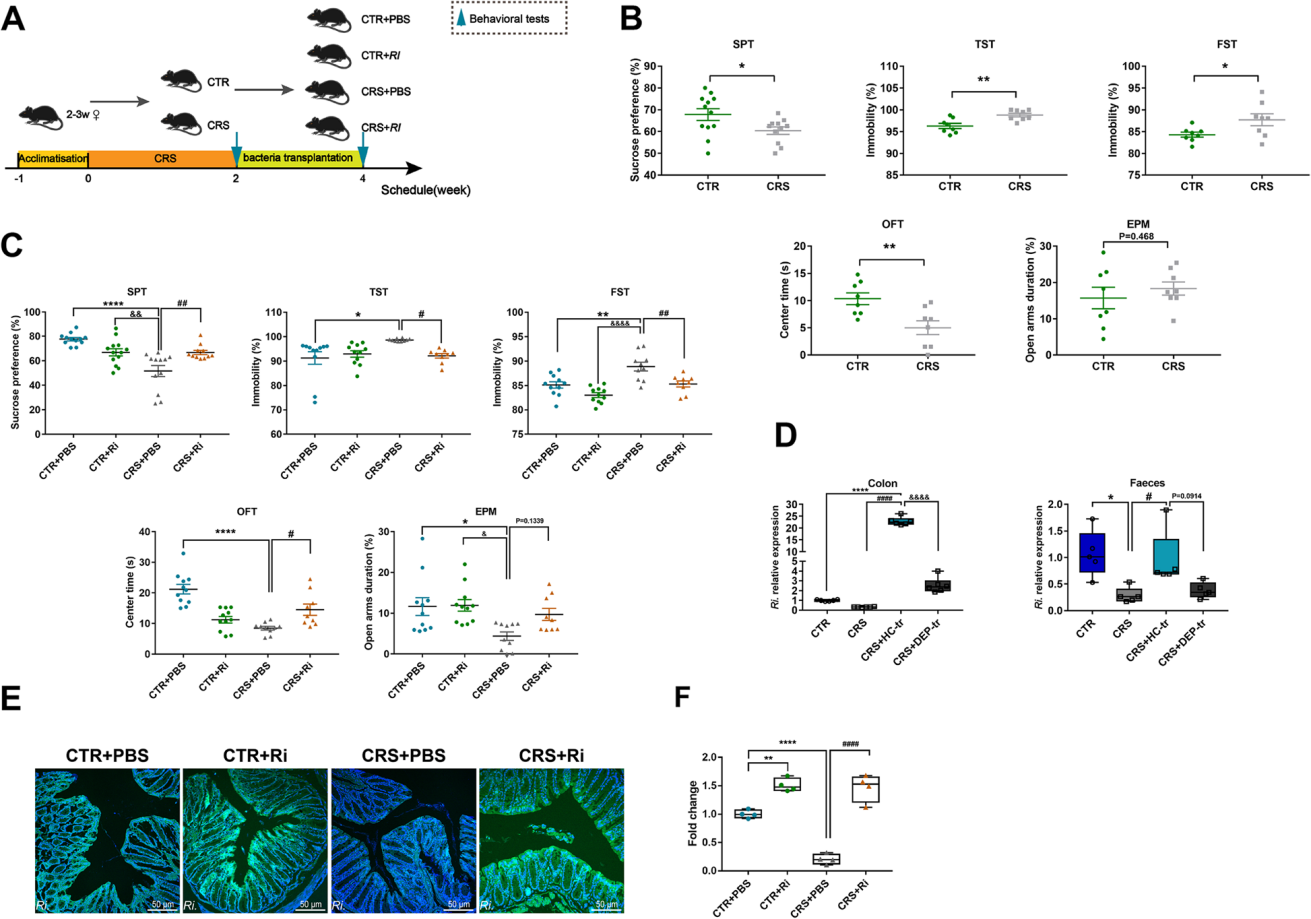
Colonic *Ri.* colonization improved depression-like behaviors in mice. **A** Protocol diagram of the time courses of the *Ri.* transplantation and behavioral tests. The mice were exposed to CRS and then the behavioral tests were conducted. Half of the CTR and CRS mice were selected for *Ri.* gavaging, while the other mice were administered equal volumes of sterile PBS. The transplantation procedure lasted for 2 weeks. **B** Establishment of the CRS-induced depressive mice model as determined through SPT, TST, FST, OFT, and EPM test assays. **C** Effects of *Ri.* on CRS-induced depressive behaviors in mice. **D** Abundance of *Ri.*in the colon and feces of the FMT mice. **E** FISH-determined *Ri.* colonization. **F** Normalized fluorescence intensity analysis of *Ri.* colonization. Scale bar 50 μm. Data were displayed as mean ± SEM. Except for the one-way ANOVA used in **D**, other significant differences were determined via Student’s *t* test or two-way ANOVA and Tukey’s multiple comparison procedure. ^*^p < 0.05, ^**^p < 0.01, ^****^p < 0.0001 *vs.* the CTR + PBS group; ^&^*p* < 0.05, ^&&^*p* < 0.01, ^&&&&^*p* < 0.0001 *vs.* the CTR + Ri group; ^#^*p* < 0.05, ^##^*p* < 0.01, ^####^*p* < 0.0001 *vs.* the CRS + PBS group

Having documented the beneficial antidepressant effects of normobiotic FMT, we hypothesized that some microbiota in healthy adolescent volunteers may function as the probiotics to alleviate depression. Using 16S sequencing (Fig. 1E, F and Figure S1B) and combination of related studies [1, 29–31], we considered *Ri*., one favorable species of the genus *Roseburia*, as the target intervention microbial strain (Fig. 8A). Surprisingly, human *Ri.* was well customized in the mouse colon after FMT, as evidenced by its strong expression in the colon and feces of CRS + HC-tr mice (Fig. 8D). To further characterize the efficient colonization of *Ri.* in the mouse colon, green fluorescence-labeled *Ri.* was applied, in accordance with the results obtained in HC-GM transferring mice, the fluorescence intensity dramatically increased in the colonic segment of adolescent mice gavaged with *Ri.* in the CTR + Ri and CRS + Ri groups, respectively, as determined by FISH analysis (Fig. 8E, F), validating the efficient colonization of *Ri.* The question was then moved to whether *Ri.* colonization has beneficial effects on depression. Intriguingly, *Ri.* transplantation dramatically attenuated the depression- and anxiety-like behaviors induced by CRS (Fig. 8B, C), suggesting that *Ri.* and *Ri.* from healthy adolescent volunteers serve a salutary action on adolescent depression.

### *Ri.* transplantation mitigates CRS-induced perturbation of neurotransmitters by the Trp-Kyn metabolic pathway

**Fig. 9:**
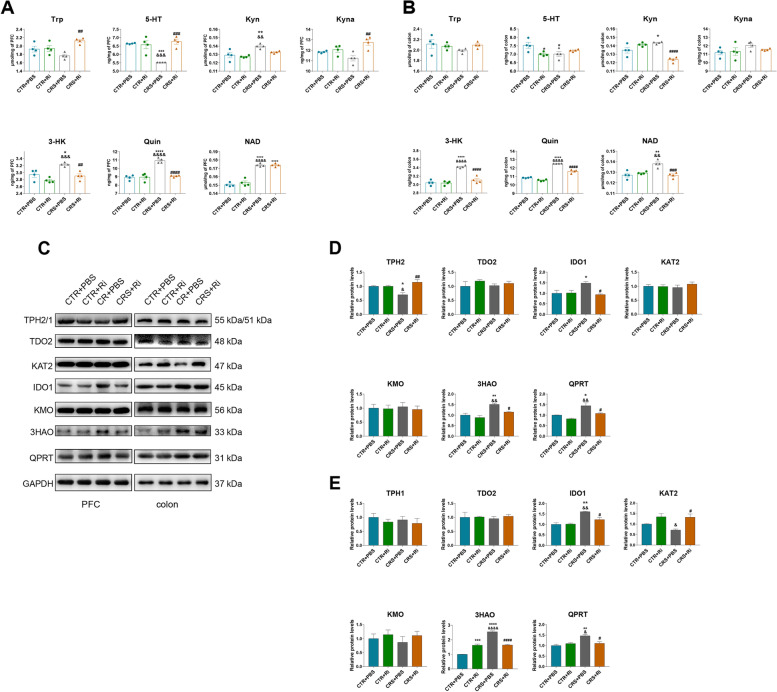
*Ri.* treatment improved the perturbation of neurotransmitters from the Trp-Kyn metabolic pathway in mouse brains and colons. **A** Levels of 5-HT and KP metabolites in the PFCs of the mice. **B** Levels of 5-HT and KP metabolites in the colons of the mice. **C** Protein expression of the rate-limiting enzymes of KP metabolism in the mice PFCs and colons. **D** The statistical plots of the PFCs. **E** The statistical plots of the colon. Data were displayed as mean ± SEM. Significant differences were determined via two-way ANOVA and Tukey’s multiple comparison procedure. ^*^p < 0.05, ^**^p < 0.01, ^***^p < 0.001, ^****^p < 0.0001 *vs.* the CTR + PBS group; ^&^*p* < 0.05, ^&&^*p* < 0.01, ^&&&&^*p* < 0.0001 *vs.* the CTR + Ri group; ^#^*p* < 0.05, ^##^*p* < 0.01, ^####^*p* < 0.0001 *vs.* the CRS + PBS group

To decipher the role of *Ri.* in attenuating CRS-induced depressive behaviors, the Trp-Kyn metabolic pathway was investigated. As depicted in Fig. 9A, B; Figure S4A and S4B, CRS conspicuously suppressed 5-HT levels and reciprocally activated KP events in the PFC, serum, and colon, manifesting as elevated levels of Kyn, 3-HK, Quin, and NAD^+^. To support these phenomena, the Trp-derived rate-limiting enzymes aligned to these Trp-Kyn metabolites were detected, and significant reductions of TPH2 in the PFC and KAT2 in the colon were observed in CRS-treated mice, along with evident elevation of IDO1, 3HAO, and QPRT in the mouse brain and gut induced by CRS (Fig. 9C–E). Importantly, *Ri*. did restore the aberrant activation of KP metabolism. To gain more mechanistic insights into CRS-induced Kyn elevation in the PFC, large neutral amino acid transporter 1 (LAT1), a key transporter responsible for delivering larger, neutral amino acids and Kyn across the blood–brain barrier (BBB) from the peripheral circulation [32–34], was examined (Figure S5A and S5B). In line with the increased level of Kyn found in PFC, LAT1 expression in the CRS mouse brain was markedly increased.

### Inoculation of *Ri.* improves CRS-induced synaptic plasticity and glial activity

**Fig. 10:**
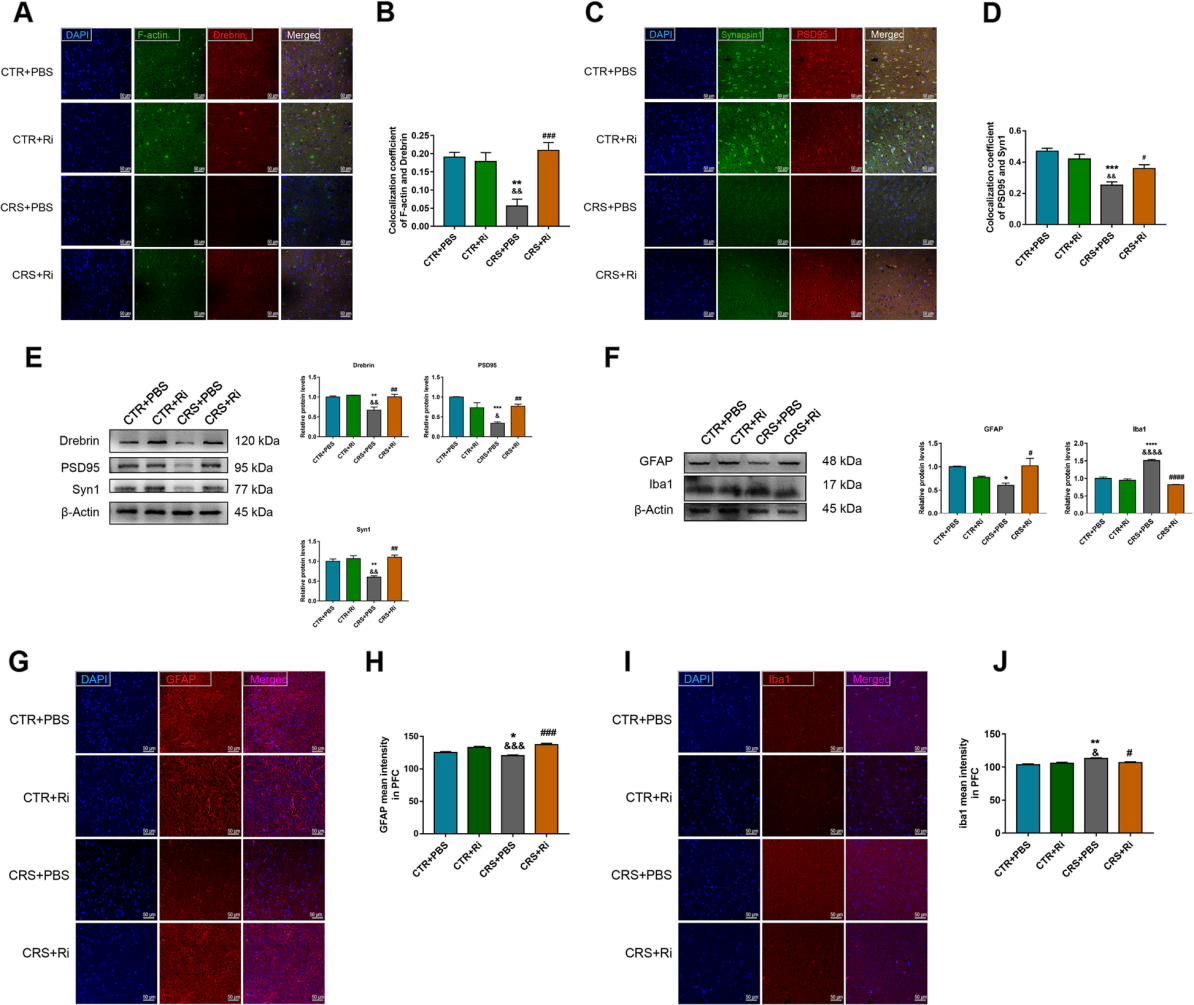
*Ri.* treatment improved synaptic plasticity and glial activities. **A**,** B** Colocalization of F-actin and Drebrin in the mice PFCs along with their statistical graphs. **C**, **D** Colocalization of Syn1 and PSD95 along with the coefficient analysis. **E** Relative protein expression of Drebrin, PSD95, and Syn1 along with their statistical graphs. **F** Protein expression of GFAP and Iba1 along with their statistical graphs. **G**, **H** MFI of GFAP and the statistical graph **H**. **I**, **J** MFI of Iba1 and the statistical graph **J**. Scale bar 50 μm. Data were displayed as mean ± SEM. Significant differences were determined via two-way ANOVA and Tukey’s multiple comparison procedure. ^*^p < 0.05, ^**^p < 0.01, ^***^p < 0.001, ^****^p < 0.0001 *vs.* the CTR + PBS group; ^&^*p* < 0.05, ^&&^*p* < 0.01, ^&&&^*p* < 0.001, ^&&&&^*p* < 0.0001 *vs.* the CTR + Ri group; ^#^*p* < 0.05, ^##^*p* < 0.01, ^###^*p* < 0.001, ^####^*p* < 0.0001 *vs.* the CRS + PBS group

Impairment of synaptic plasticity is one of the main causes of depression. To elucidate the mechanisms underlying the protective effects of *Ri.* on CRS-induced depression, synaptic markers and glial activities were investigated. As displayed in Fig. 10A–E, CRS induction decreased the densities of dendritic spines and synapses. This was significantly reversed by *Ri.* inoculation, as CRS induced downregulation of Drebrin, PSD95, and Syn1 was markedly rescued by *Ri.* administration. Similar to the results of healthy adolescent microbiota transplantation, aberrantly activated microglial cells and activity-suppressed astrocytes were ameliorated by *Ri*. transplantation, manifesting as decreased Iba-1 and increased GFAP expression (Fig. 10F–J), demonstrating the salutary effects of *Ri*. in anti-depression.

### *Ri.* exerts beneficial functions on colon barrier of CRS mice

**Fig. 11:**
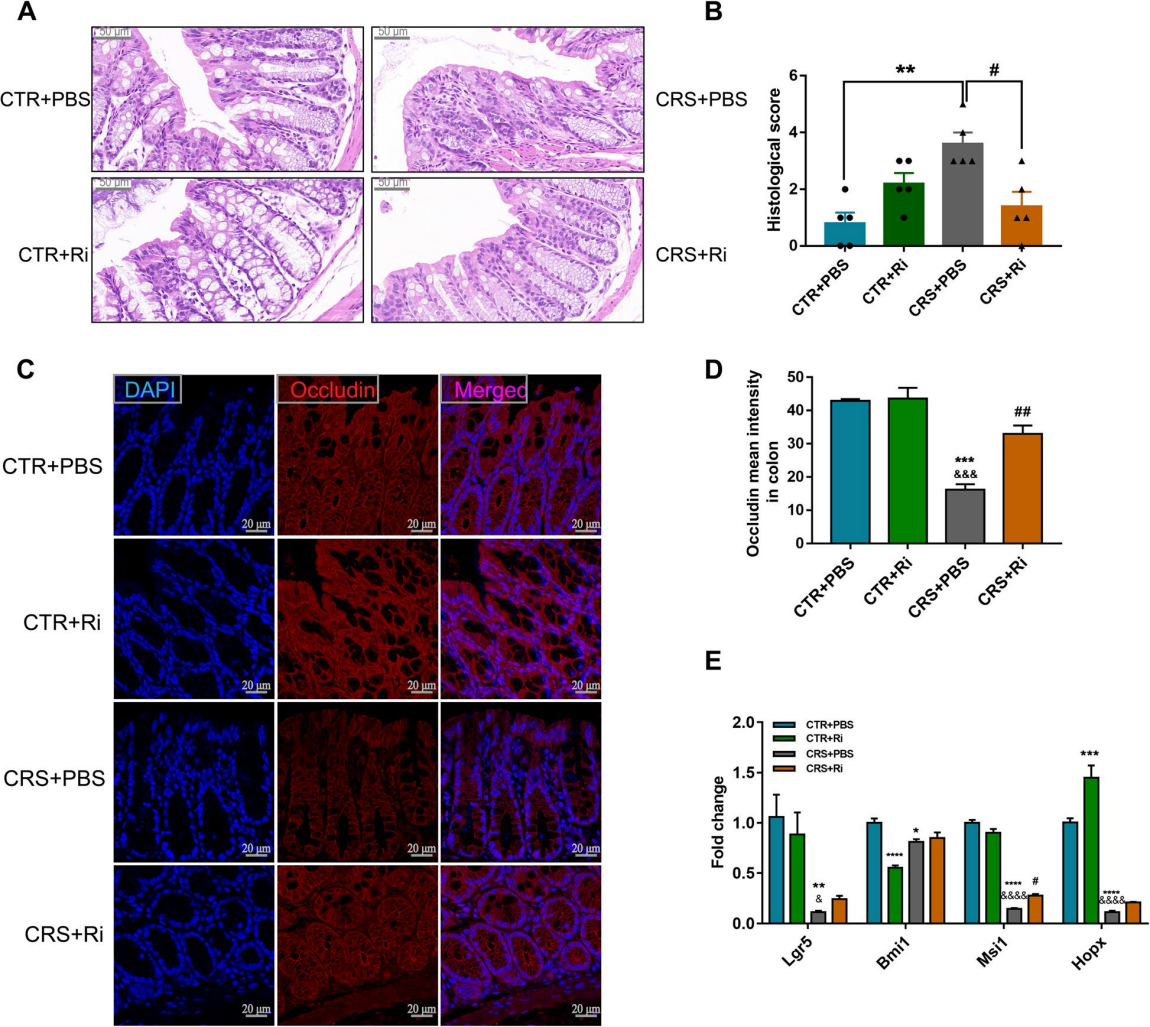
*Ri.* treatment reversed colon barrier impairment in CRS mice. **A**, **B** H&E staining of the mice colons (scale bar 50 μm) along with the histological score analysis. **C**, **D** IF images **C** and MFI analysis **D** of Occludin; scale bar 20 μm. **E** mRNA expression of ISC markers. Data were displayed as mean ± SEM. Significant differences were determined via two-way ANOVA and Tukey’s multiple comparison procedure. ^*^p < 0.05, ^**^p < 0.01, ^***^p < 0.001, ^****^p < 0.0001 *vs.* the CTR + PBS group; ^&^*p* < 0.05, ^&&&^*p* < 0.001, ^&&&&^*p* < 0.0001 *vs.* the CTR + Ri group; ^#^*p* < 0.05, ^##^*p* < 0.01 *vs.* the CRS + PBS group

The relative abundance of *Ri.* was significantly decreased in inflammatory bowel disease (IBD) patients [35] which may be ascribed to its role in maintaining intestinal epithelial integrity and anti-inflammation [31]. Intestinal epithelial cells (IECs) serve as a physical and antimicrobial barrier against the microbiota, therefore, based on the protective effects of GM transplantation from healthy adolescents on colon endothelial cells, we wondered whether *Ri*. protects the structural integrity of the barrier in CRS mice. As expected, compared with the control mice, the epithelial integrity was significantly disrupted in the CRS mice, manifesting as increased crypt atrophy and lymphocyte infiltration at the base of the crypt and mucosal base (Fig. 11A, B). These changes were concomitant with decreased amounts of crypts and Occludin, which serves as one marker for barrier integrity (Fig. 11C, D). Of note, *Ri.* transplantation obviously mitigated CRS-induced intestinal epithelial impairment (Fig. 11A–D).

Epithelial regeneration is essential for barrier maintenance and organ function [36, 37]. Therefore, specific markers of intestinal stem cells (ISCs) were investigated. As depicted in Fig. 11E, CRS led to significantly decreased mRNA expression of ISCs, while *Ri*. intervention resulted in a pronounced elevation of *MSI1* expression and slightly increased *LGR5*, *BMI1*, and *HOPX* levels, suggesting a favorable resolution of *Ri.* colonization in the colon and fewer effects on the neogenesis of colonic epithelial cells.

## Discussion

Depression is one of the leading causes of death in adolescents. Most affected adults have their first depressive episode during adolescence, highlighting the need for early identification and treatment [5]. Therefore, a better understanding of the underlying mechanisms associated with adolescent depression is essential to improve the efficacy of available treatments. In the current study, we assumed that perturbation of both brain- and colon-derived Trp metabolism is implicated in CRS induced depression like changes in mice, while the screened target microbiota *Ri.* exerts the anti-depression effects by restraining the toxic metabolite generation by ameliorating the Trp metabolism towards the Kyn metabolic pathway. The present study demonstrated that perturbation of the Trp-Kyn metabolic pathway is associated with the gut microbiota alteration in adolescent depression, these findings specifically extended the knowledge of the potential roles of the target gut microbiota *Ri.* in terms of the modulation of the Trp-Kyn metabolic pathway and the potential “cross talk” along the microbiota-gut-brain axis induced by CRS. To our knowledge, this is, for the first study to reveal the adolescent depression through neurotransmitter perturbation in the Trp-Kyn metabolic pathway. In addition, we addressed the beneficial effects of the targeted microbiota screened out from the healthy adolescent microbiome on improving Trp-Kyn metabolism, synaptic plasticity, and depressive behaviors, which may provide promising biomarkers for diagnosis, prognosis evaluation and treatment of adolescent depression.

Mounting evidence suggests that probiotic consumption during adolescence attenuates LPS-induced neuroinflammation and protects against depression-like behaviors in adulthood, which may be linked to reversal of inflammation-induced gut dysbiosis, thereby decreasing one’s vulnerability to brain disorders later in life [38, 39]. Given the prominent roles of microbial homeostasis in adolescence, 16S rRNA sequencing analysis showed a decrease in *Firmicutes* in depressed youths and this effect could be restored by sertraline. Accordingly, specific genera belonging to this phylum, such as *Faecalibacterium*,* Blautia*,* Phascolarctobacterium*, and *Roseburia* strongly enriched in HC or DEP-sertraline-treated adolescents, which indicated the potential sensitivity of these bacterial genera to alterations of the 5-HT levels. Notably, these flora are capable of generating short-chain acids (SCFAs) [17, 37, 40–42], an important energy source for colon epithelial cells [43] and energy substrates [44]. Moreover, it has been reported that SCFAs per se plays a vital role in anti-neuroinflammation [45], maintaining BBB integrity [46], and influencing cognition and mood [47] through the MGB axis [48–50].

Early in 1969, the KP hypothesis was proposed to explain the depression [51], in which the paucity of 5-HT is said to have resulted from a more active shift from Trp to Kyn, thereby weakening the suppression of cortisol generation. The increased cortisol ulteriorly activates the enzyme catalyzing KP metabolism, and consequently, a vicious circle of 5-HT paucity is formed. This concept was then improved on by evidence of the neuroactivity of KP metabolites; for example, Quin, an excitotoxic agonist of the N-methyl-D-aspartic acid receptor (NMDAR) [52], is involved in at least nine mechanisms of neurotoxicity, including ROS production, BBB damage, cytoskeleton stability disruption, tau phosphorylation increase, and autophagy impairment [10]. Moreover, 3-HK, the upstream substance of Quin, not only traverses the BBB, but also induces myriad free radicals [53] and is involved in the inhibition of complex I, II, and IV in the electron transport chain (ETC), thereby aggravating mitochondrial impairment and intracellular oxidative stress responses [54]. In contrast, Kyna, a metabolite of another branch of the KP, has been shown to be neuroprotective [55], due to its antagonistic role on NMDAR and alpha7 (α7) nicotinic acetylcholine receptors (see the Trp-derived metabolic pathway in Figure S2A) [56].

By detecting the monoamine neurotransmitters and Trp metabolites in the serum and urine of adolescent depressive patients, we found that 5-HT (*r* =  − 0.520, *p* = 0.006), Trp (*r* =  − 0.210, *p* = 0.296), Kyn (*r* =  − 0.390, *p* = 0.047) and Kyna (*r* =  − 0.540, *p* = 0.003) concentrations in serum were negatively associated with RCADS-25 scores. Meanwhile, Kyna levels had the highest negative correlation with the scale scores, demonstrating its effects as a potential biomarker for prognosis evaluation in depression interventions. These findings strongly support the recent cross-sectional studies of the major depressive disorder (MDD) population, stating that neurotransmitters from the Trp-Kyn metabolic pathway, including Trp, Kyn, and Kyna, may serve as potential biomarkers that facilitate the diagnosis and prediction of depression treatment [57–60]. In the present study, we found that Quin, a toxic metabolite generated by the rate-limiting enzyme 3HAO, obviously decreased in both serum and urine after sertraline administration, accurately reflecting its antidepressant effects. This result will ideally provide the novel insights into the efficacy of selective serotonin reuptake inhibitors (SSRIs) and the potential drivers of depression in adolescence.

The intestinal microbiota dysbiosis is associated not only to depression, but also to autism and anxiety [61, 62]. Gut dysbiosis and neurotransmitter metabolism perturbation simultaneously occur in mice with depression- and anxiety-like behaviors [14], which may be closely linked with host behaviors and pathologies. Although elaborately explained the roles of the brain derived neurotransmitter in terms of their neuroactivities, the function of KP metabolites in the gastrointestinal (GI) tract still remains poorly understood [56]. To unravel the underlying relationships among KP metabolism, microbial composition and depression development, a juvenile chronic stress murine depression model was established. By performing FMT, the GM from HC adolescents remarkably ameliorated depressive behavioral phenotypes and alleviated the abnormal conversion from Trp to Kyn in both brain and colon. Specifically, inhibition of the increased toxic metabolites, such as 3-HK, 3-HAA, and Quin, along with decreased pivotal rate-limiting enzymes (IDO1 and 3HAO) was observed. Taken together, this evidence implies that some probiotics may rescue a host’s normal behaviors and Trp-Kyn homeostasis functions. Surprisingly, the expression of QRPT and NAD^+^ was significantly increased in the brains and colons of the CRS mice, which was not consistent with the current reports on neurodegenerative diseases and aging models [63, 64]. We suspect that these expression levels may pivot during early-stage responses to myriad precursors of NAD^+^ in its a de novo pathway, detailly, catalyzing excessive toxic Quin to non-toxic NAD^+^ by QPRT in responsible to stress, and less consumption, resulting in an increase level of NAD^+^, which provides us the future exploration on expression and function of the NAD^+^ consumers, such as sirtulins, PARP and CD38 [65]. Moreover, the microbiomes from DEP adolescents that were transferred into CRS mice did not lead to more aggravated depressive behaviors and KP metabolic fluctuations compared to in CRS-treated mice, thus highlighting the beneficial roles of the normobiotic microbial ecology in maintaining psychological behaviors [66].

Reductions in presynaptic, postsynaptic, and dendritic spine numbers are prominent characteristics of depression [67, 68].In recent studies, imbalances in neurotransmitters were reported to be associated with synaptic injuries: for example, in synaptic injury cases, the KP downstream toxic metabolite Quin is abnormally elevated and is capable of inducing neuron atrophy, which then leads to a reduction in neurite outgrowth [22]. Furthermore, as the principal source for the generation of Quin and 3-HK [26], activated microglia are responsible for the engulfment of neural dendritic spines, leading to more severe neuronal atrophy and inflammation in adolescent depression [69]. Reciprocally, astrocytes, exert their protective effects via Kyna synthesis and counteract the excitotoxicity of Quin [26]. In the CRS mouse model, after HC microbiota transplantation, the densities of dendritic spines and synapses were strikingly enhanced, and these were accompanied by suppressed microglia and activated astrocytes. This indicates that the normobiotic microbiota may exert its direct or indirect effects via a new way related to maintaining enterogenous KP stability and the metabolites that can cross the BBB, resulting in balancing KP metabolism, restoring synaptic plasticity, and ultimately improving depression-like behaviors.

Leaky gut may be one of the underlying causes of illnesses involving concomitant downstream injuries [27]. In its pathological progression, bacterial structural toxins and toxic metabolites in the lumen are more likely to leak into the peripheral circulation, thereby influencing the peripheral system and CNS through the impaired intestinal barrier [70]. Of note, these toxins are more commonly found in IBD and celiac disease (CD) concomitant with depression [27]. Consistently, in the current study, the elevated expression of IDO1 induced by CRS facilitated the conversion of the Trp signaling to the toxic Kyn pathway, contributing to Kyn, 3-HK, and Quin accumulation in colon, which may aggravate gut integrity impairment, and increase periphery Kyn and other toxic metabolites, serving as an accomplice of depression. Notably, HC-GM transplantation effectively reversed these adverse effects, highlighting the benefits of some components in HC flora.

In the light of the target bacteria screened in the studied population, we investigated whether some of the potential probiotic species of the HC flora play an efficient role in ameliorating depressive phenotypes. Therefore, *Ri.*, an anaerobic bacterium producing butyric acid with anti-inflammatory effects in IBD, was screened out from the comparison among the healthy adolescent volunteers, unmedicated depressive adolescents and the sertraline-treated patients. Noteworthily, after FMT, *Ri.* effectively colonized the mouse colon, thereby ameliorating depression-like behaviors and maintaining KP metabolism homeostasis. Mechanistically, decreased levels of Kyn and Quin that were induced by *Ri.* were identified via inhibition of the key enzymes IDO1 and 3HAO in mouse brain, respectively. Of note, the CNS receives about 60% of its Kyn from the periphery via transport across the BBB [71], where, under inflammatory conditions, the LAT1 plays a crucial role [10]. In this study, *Ri.* transplantation obviously ameliorated the high levels of Kyn that were induced by CRS, and apart from blocking IDO1 expression in the brain, decreased LAT1 expression levels mediated by *Ri.* may also exert pivotal functions in reduction of Kyn in the brain, thereby, ameliorating the levels of the toxic metabolites. Though Trp can be also transported by LAT1, in the current study, the levels of Trp were not significantly changed in control and CRS group, this may ascribe to the increased conversion from Trp to Kyn and decreased shift from Trp to 5-HT, as evidenced by the up-regulated expression of IDO1 and down-regulated one of TPH2 in CRS group, respectively. In addition, enhanced levels of 5-HT in the brain after *Ri*. colonization may be attributed to the promotion of TPH2 expression. Strikingly, *Ri.* exhibited a salutary effect on the mouse brain, manifesting as an improvement in synaptic plasticity, inhibition of microglial activation and an increase in astrocyte activity. Moreover, *Ri.* also maintained the integrity of the colon barrier in CRS mice which may act on tight junction proteins via its product, butyric acid [72] or via self-structural substances [73]. Taken together, our results demonstrate the possibility of *Ri*. transplantation as a depression treatment and decipher the underlying mechanisms of Trp-Kyn metabolism along the MGB axis. However, we cannot rule out the possible involvement of the vagus nerve in connection of the microbiota and the brain. One recent report indicated FMT from the α7 subtype of the nicotinic acetylcholine receptor knockout mice (Chrna7 KO mice) showed depression-like phenotypes treated with an antibiotic cocktail, while subdiaphragmatic vagotomy significantly blocked the development of depression-like phenotypes [74], it is possible that some metabolites of the *Ri.* may participate in stimulation of the vagal afferent nerves, modulating Trp-5-HT and Trp-Kyn metabolism in the brain. Therefore, more studies are reasonable to be performed in this field.

### Limitations of the study

The present study has some limitations. First, due to the COVID-19 epidemic, the collection of population samples could not be conducted smoothly, thus the sample size is small and it will need to be amplified in the future. Second, the causalities among synaptic plasticity and KP, colon barrier integrity, and KP should be validated with stronger supports, for example, the usage of IDO1 and 3HAO knockout mice. Third, the most efficient compositions of the *Ri.* need to be clarified, and proteomics testing for bacterial secretions or structural substances is urgently needed in the future.

## Conclusions

**Fig. 12:**
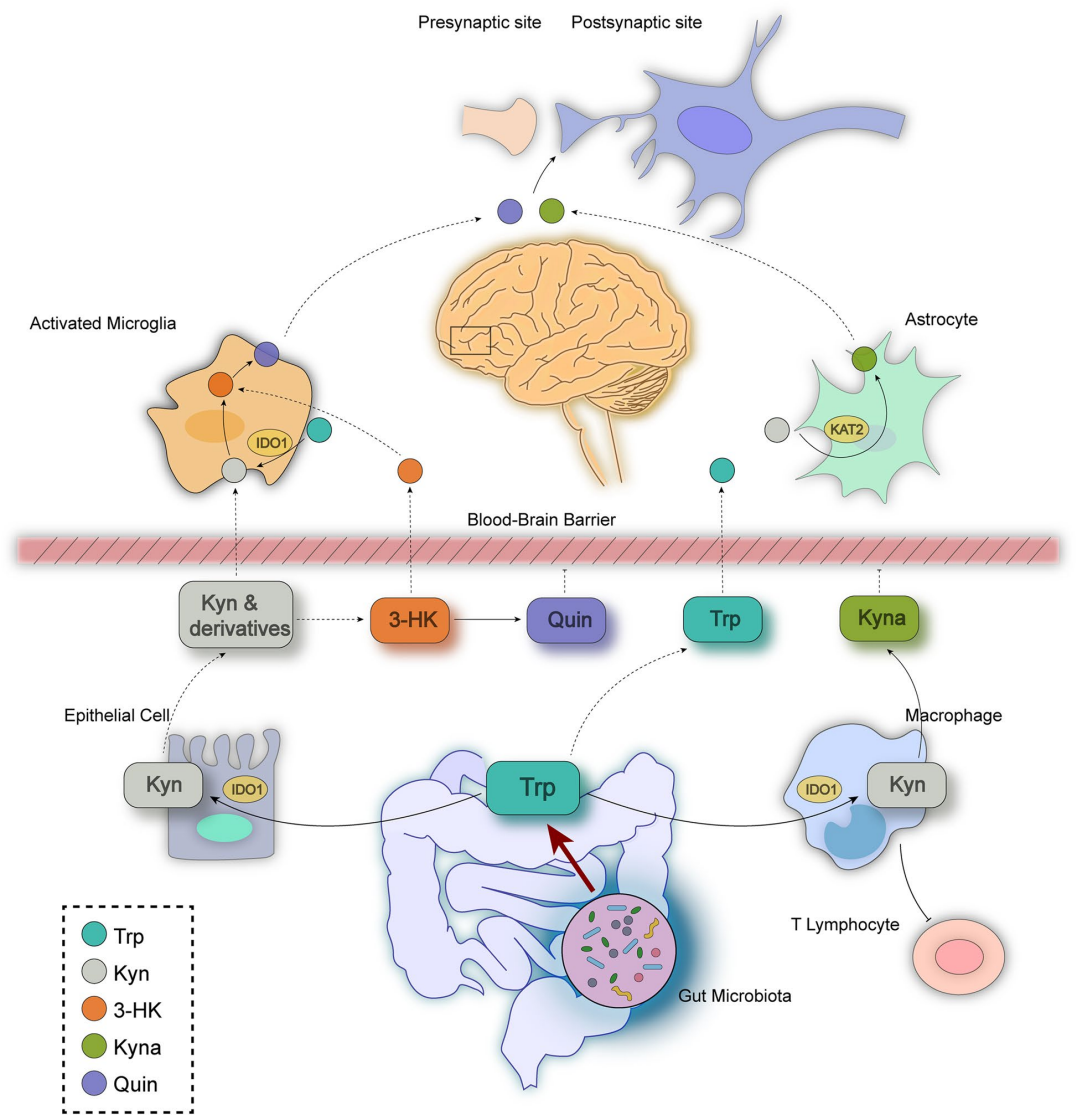
A schematic depicting the roles of the gut microbiota in CRS-induced depression-like behavioral changes along the microbiota-gut-brain axis. The abundance of *Ri.*is rich in healthy adolescents, decreased in adolescents with depression, and reversed after sertraline treatment. The GM (from the feces of healthy adolescents and consisting of *Ri.*) exerts beneficial effects by ameliorating CRS-induced perturbation of the Trp-Kyn metabolic pathway. These effects are characterized by the increased conversion of Trp to 5-HT, and reciprocally, decreased toxic metabolite levels (Quin and 3-HK) that mechanistically unravel as the levels of TPH2/1 and KAT2 increase, along with decreased expression of IDO1 and 3HAO. The ameliorated colonic epithelial cell impairment combined with the improved KP metabolism driven by *Ri.* facilitated the pronounced protection of synaptic plasticity and improved glial activities, thus providing a novel therapeutic strategy for depression intervention

In conclusion, *Roseburia* was screened out from the healthy adolescent volunteers due to its fair sensitivity and specificityin depression prediction. FMT of healthy adolescent volunteers or transplantation of the pure *Ri*. inhibited the expression of the rate-limiting enzymes (IDO-1, 3-HAO etc.) and reversed the CRS induced conversion from Trp to Kyn in both brain and colon. Additionally, *Ri*. administration improved the gut barrier integrity in the colon where the toxic metabolites (Kyn, Quin and 3-HK) accumulated, and decreased transport of the Kyn from the periphery to the brain, thereby improved synaptic plasticity and glial activity, and displayed anti-depression functions (Fig. 12).

## Supplementary Information

**Additional file 1: Figure S1. **Gut microbiota (GM) composition differences among healthy adolescent controls (HC), unmedicated depressive adolescents (DEP) and sertraline-treated adolescents. **A** Venn diagram of feature profiling among three groups. **B** Genus abundance of *Roseburia* in the HC, DEP, and DEP-sertraline treated groups. Data were displayed as Minimum to Maximum in **B**. Significant differences among the three groups were determined via Kruskal–Wallis test, Benjamini–Hochberg test was applied for multiple comparison. **Figure S2. **The detailed Trp-Kyn metabolic pathway and NAD^+^ concentration in serum. **A** Representation of Trp catabolism along 5-HT and Kyn branches. **B** NAD^+^ concentration in the serum of DEP and DEP-sertraline treated adolescents. **C** Correlation analysis between serum NAD^+^ level and RCADS. Data were represented as mean ± SEM. ^****^p < 0.0001 versus DEP group. Significant differences were determined via Student’s *t*-test and correlations between RCADS and NAD^+^ levels was shown in Pearson’s r value. Trp, tryptophan; 5-HT, 5-hydroxytryptamine; TDO: tryptophan 2,3-dioxygenase; IDO: indoleamine 2,3-dioxygenase; Kyn: Kynurenine; KATs: kynurenine aminotransferases; Kyna: kynurenic acid; KYNU: kynureninase; AA: anthranilic acid; KMO: kynurenine 3-monooxygenase; 3-HK: 3-hydroxycanuridine; XA: xanthurenic acid; 3-HAA: 3-hydroxyanthranilic acid; PA: picolinic acid; 3HAO: 3-hydroxyanthranilate 3,4-dioxygenase; Quin: quinolinic acid; QPRT: quinolinate phosphoribosyl transferase; NAM: nicotinamide; NAD/NADP: nicotinamide adenine dinucleotide/nicotinamide adenine dinucleotide phosphate. RCADS: the Revised Child Anxiety and Depression Scale. **Figure S3.** Trp-derived metabolites in the PFC and serum of FMT mice. **A** Other downstream products of kynurenine pathway (KP) and Trp-5-HT branch in PFC determined by UHPLC-MS/MS. **B** Levels of Trp-Kyn and Trp-5-HT pathway metabolites detected by UHPLC-MS/MS in mouse serum of GM transplantation model, and the levels of Kyna, Quin and NAD^+^ were measured by ELISA. Data were represented as mean ± SEM. ^*^p < 0.05, ^**^p < 0.01, ^***^p < 0.001, ^****^p < 0.0001 versus CTR group; ^##^*p* < 0.01, ^####^*p* < 0.0001 versus CRS group; ^&&&^*p* < 0.001, ^&&&&^*p* < 0.0001 versus CRS + HC-tr group. All data here were analyzed by one-way ANOVA, Tukey’s multiple comparison test was applied for post hoc test. **Figure S4. **Concentrations of neurotransmitters along Trp-derived metabolic pathways in *Ri*. intervened mice. **A** Other downstream products of KP and Trp-5-HT branches in PFC. **B** Concentrations of neurotransmitters from Trp-Kyn and Trp-5-HT metabolic pathway in mouse serum. Data were represented as mean ± SEM. ^*^p < 0.05, ^**^p < 0.01, ^***^p < 0.001, ^****^p < 0.0001 versus CTR + PBS group; ^&^*p* < 0.05, ^&&^*p* < 0.01, ^&&&^*p* < 0.001, ^&&&&^*p* < 0.0001 versus CTR + Ri group; ^#^*p* < 0.05, ^##^*p* < 0.01, ^####^*p* < 0.0001 versus CRS + PBS group. All data here were analyzed by two-way ANOVA, Tukey’s multiple comparison test was applied for post hoc test. **Figure S5. **Expression of large neutral amino acid transporter 1 (LAT1) in PFC and mucus changes in colon of each treatment group. **A** LAT1 expression in PFC of *Ri*. gavage mice. **B** Statistical graph of LAT1. Data were represented as mean ± SEM. ^*^p < 0.05, versus the CTR + PBS group; ^#^*p* < 0.05 versus the CRS + PBS group. Significant differences were determined via two-way ANOVA, Tukey’s multiple comparison test was applied for post hoc test. **Table S1.** Demographic characteristics of depressive and healthy control participants. **Table S2.** Specific primers sequences.

**Additional file 2.**

## Data Availability

The raw reads were deposited into the NCBI Sequence Read Archive (SRA) (BioProject ID: PRJNA894780). All data were available upon request from the authors.

## Abbreviations

MGB axis: Microbiota-gut-brain axis
KP: Kynurenine pathway
GM: Gut microbiota
Trp: Tryptophan
Quin: Quinolinic acid
CRS: Chronic restraint stress
SSRIs: Selective serotonin reuptake inhibitors
DSM-V: Diagnostic and Statistical Manual of Mental Disorders 5
RCADS-25: Revised Child Anxiety and Depression Scale-25
Kyna: Kynurenic acid
FMT: Fecal microbiota transplantation
MDD: Major depressive disorder
CTR: Control
SPT: Sucrose preference test
TST: Tail suspension test
FST: Forced swim test
OFT: Open field test
EPM: Elevated plus maze
PFC: Prefrontal cortex
MFI: Mean fluorescence intensity
CNS: Central nervous system
SCFAs: Short chain acids
BBB: Brain-blood barrier
NMDAR: N-methyl-D-aspartic acid receptor
ROS: Reactive oxygen species
ETC: Electron transport chain
IBD: Inflammatory bowel disease
