# Supplementary material for: Microbiome and tryptophan metabolomics analysis in adolescent depression: roles of the gut microbiota in the regulation of tryptophan-derived neurotransmitters and behaviors in human and mice
Source: Microbiome. 2023 Jun 30;11:145. doi: 10.1186/s40168-023-01589-9 (PMC10311725; doi:10.1186/s40168-023-01589-9)
Supplement: Supplementary file 2 — Additional file 1: Figure S1. Gut microbiota (GM) composition differences among healthy adolescent controls (HC), unmedicated depressive adolescents (DEP) and sertraline-treated adolescents. A Venn diagram of feature profiling among three groups. B Genus abundance of Roseburia in the HC, DEP, and DEP-sertraline treated groups. Data were displayed as Minimum to Maximum in B. Significant differences among the three groups were determined via Kruskal–Wallis test, Benjamini–Hochberg test was applied for multiple comparison. Figure S2. The detailed Trp-Kyn metabolic pathway and NAD+ concentration in serum. A Representation of Trp catabolism along 5-HT and Kyn branches. B NAD+ concentration in the serum of DEP and DEP-sertraline treated adolescents. C Correlation analysis between serum NAD+ level and RCADS. Data were represented as mean ± SEM. ****p < 0.0001 versus DEP group. Significant differences were determined via Student’s t-test and correlations between RCADS and NAD+ levels was shown in Pearson’s r value. Trp, tryptophan; 5-HT, 5-hydroxytryptamine; TDO: tryptophan 2,3-dioxygenase; IDO: indoleamine 2,3-dioxygenase; Kyn: Kynurenine; KATs: kynurenine aminotransferases; Kyna: kynurenic acid; KYNU: kynureninase; AA: anthranilic acid; KMO: kynurenine 3-monooxygenase; 3-HK: 3-hydroxycanuridine; XA: xanthurenic acid; 3-HAA: 3-hydroxyanthranilic acid; PA: picolinic acid; 3HAO: 3-hydroxyanthranilate 3,4-dioxygenase; Quin: quinolinic acid; QPRT: quinolinate phosphoribosyl transferase; NAM: nicotinamide; NAD/NADP: nicotinamide adenine dinucleotide/nicotinamide adenine dinucleotide phosphate. RCADS: the Revised Child Anxiety and Depression Scale. Figure S3. Trp-derived metabolites in the PFC and serum of FMT mice. A Other downstream products of kynurenine pathway (KP) and Trp-5-HT branch in PFC determined by UHPLC-MS/MS. B Levels of Trp-Kyn and Trp-5-HT pathway metabolites detected by UHPLC-MS/MS in mouse serum of GM transplantation model, and the levels of Kyna, Quin and NAD+ [file 40168_2023_1589_MOESM1_ESM.docx]

**Microbiome and tryptophan metabolomics analysis in adolescent depression: Roles of the gut microbiota in the regulation of tryptophan- derived neurotransmitters and behaviors in human and mice**

***Supplementary Information***

**Supplemental figures and legends**

**
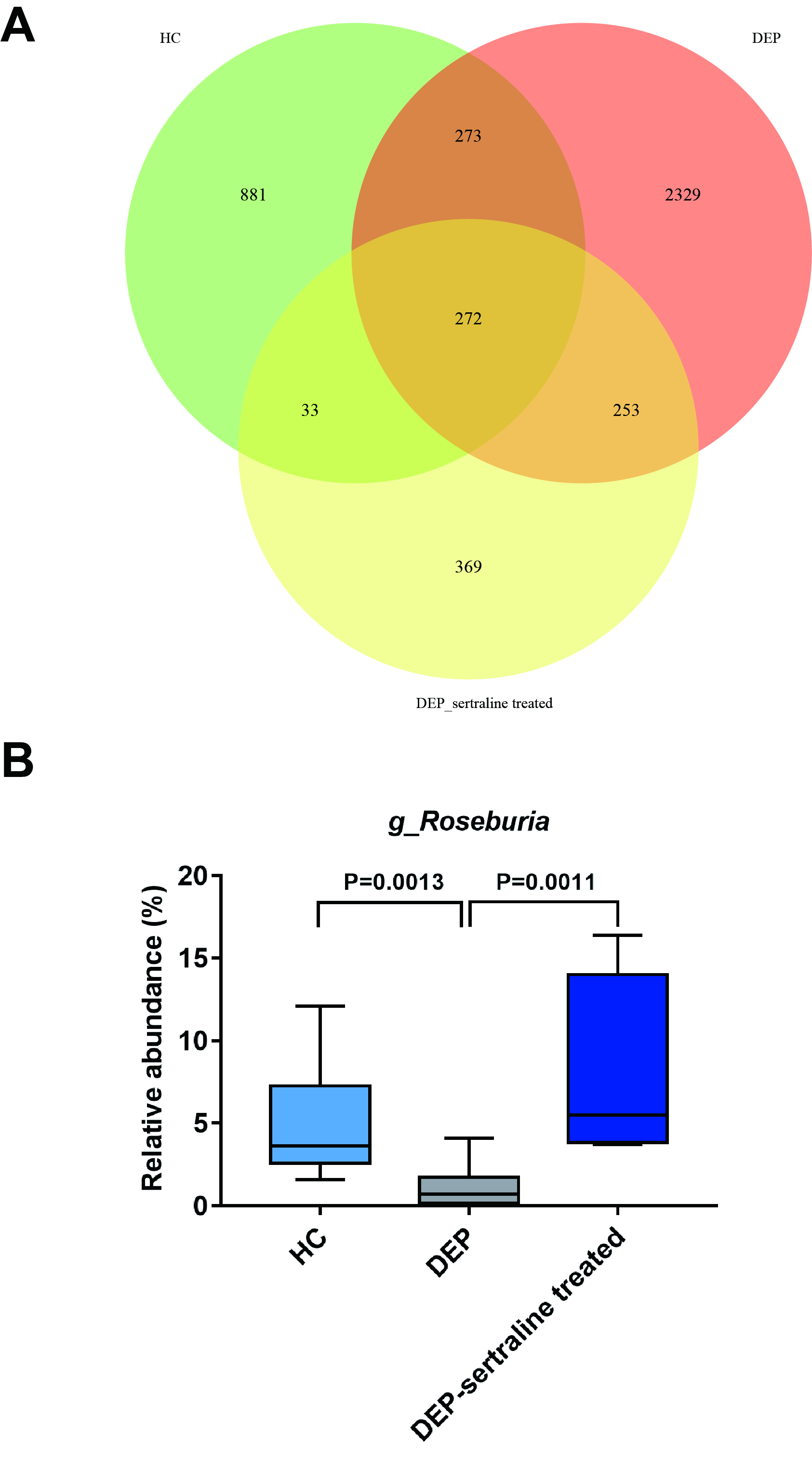
**

**Figure S1**. **Gut microbiota (GM) composition differences among healthy adolescent controls (HC), unmedicated depressive adolescents (DEP) and sertraline-treated adolescents. (A)** Venn diagram of feature profiling among three groups. **(B)** Genus abundance of *Roseburia* in the HC, DEP, and DEP-sertraline treated groups. Data were displayed as Minimum to Maximum in (B). Significant differences among the three groups were determined via Kruskal-Wallis test, Benjamini-Hochberg test was applied for multiple comparison.


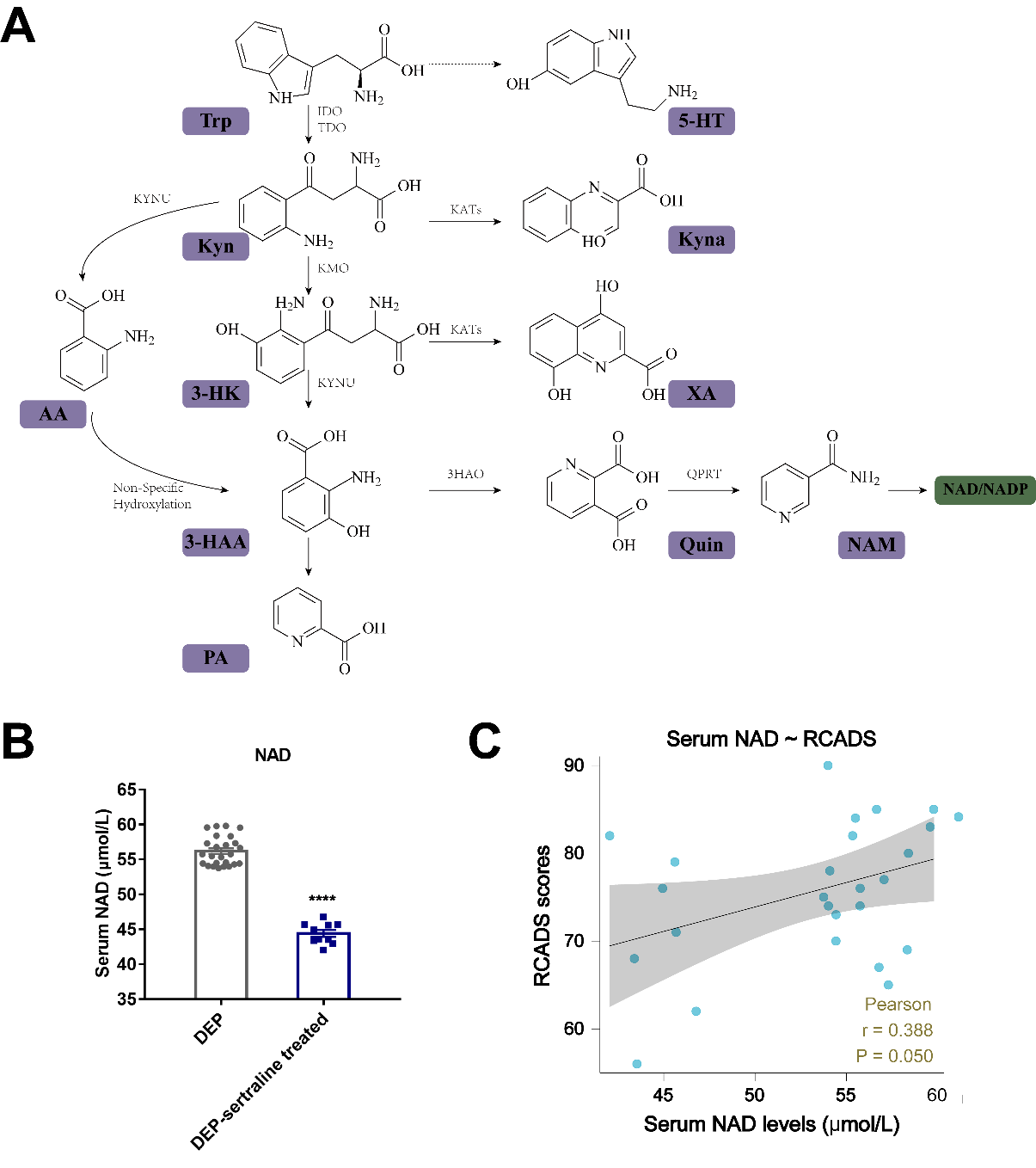


**Figure S2**. **The detailed Trp-Kyn metabolic pathway and NAD^+^ concentration in serum. (A)** Representation of Trp catabolism along 5-HT and Kyn branches. **(B)** NAD^+^ concentration in the serum of DEP and DEP-sertraline treated adolescents. **(C)** Correlation analysis between serum NAD^+^ level and RCADS. Data were represented as mean±SEM. ^****^p<0.0001 versus DEP group. Significant differences were determined via Student’s *t*-test and correlations between RCADS and NAD^+^ levels was shown in Pearson’s r value.

Trp, tryptophan; 5-HT, 5-hydroxytryptamine; TDO: tryptophan 2,3-dioxygenase; IDO: indoleamine 2,3-dioxygenase; Kyn: Kynurenine; KATs: kynurenine aminotransferases; Kyna: kynurenic acid; KYNU: kynureninase; AA: anthranilic acid; KMO: kynurenine 3-monooxygenase; 3-HK: 3-hydroxycanuridine; XA: xanthurenic acid; 3-HAA: 3-hydroxyanthranilic acid; PA: picolinic acid; 3HAO: 3-hydroxyanthranilate 3,4-dioxygenase; Quin: quinolinic acid; QPRT: quinolinate phosphoribosyl transferase; NAM: nicotinamide; NAD/NADP: nicotinamide adenine dinucleotide/nicotinamide adenine dinucleotide phosphate. RCADS: the Revised Child Anxiety and Depression Scale.


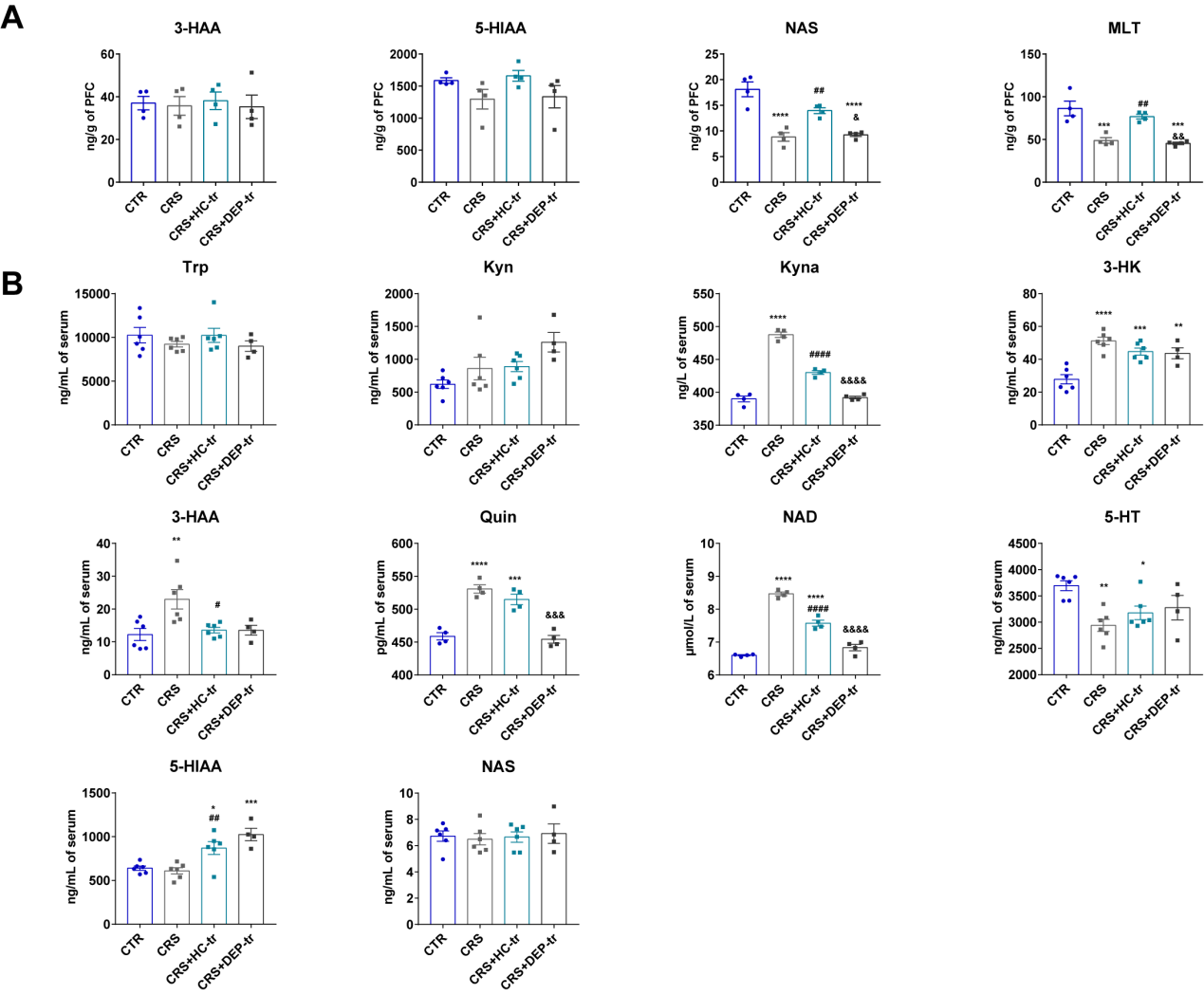


**Figure S3. Trp-derived metabolites in the PFC and serum of FMT mice. (A)** Other downstream products of kynurenine pathway (KP) and Trp-5-HT branch in PFC determined by UHPLC-MS/MS. **(B)** Levels of Trp-Kyn and Trp-5-HT pathway metabolites detected by UHPLC-MS/MS in mouse serum of GM transplantation model, and the levels of Kyna, Quin and NAD^+^ were measured by ELISA.

Data were represented as mean±SEM. ^*^p<0.05, ^**^p<0.01, ^***^p<0.001, ^****^p<0.0001 versus CTR group; ^##^p<0.01, ^####^p<0.0001 versus CRS group; ^&&&^p<0.001, ^&&&&^p<0.0001 versus CRS+HC-tr group. All data here were analyzed by one-way ANOVA, Tukey’s multiple comparison test was applied for post hoc test.


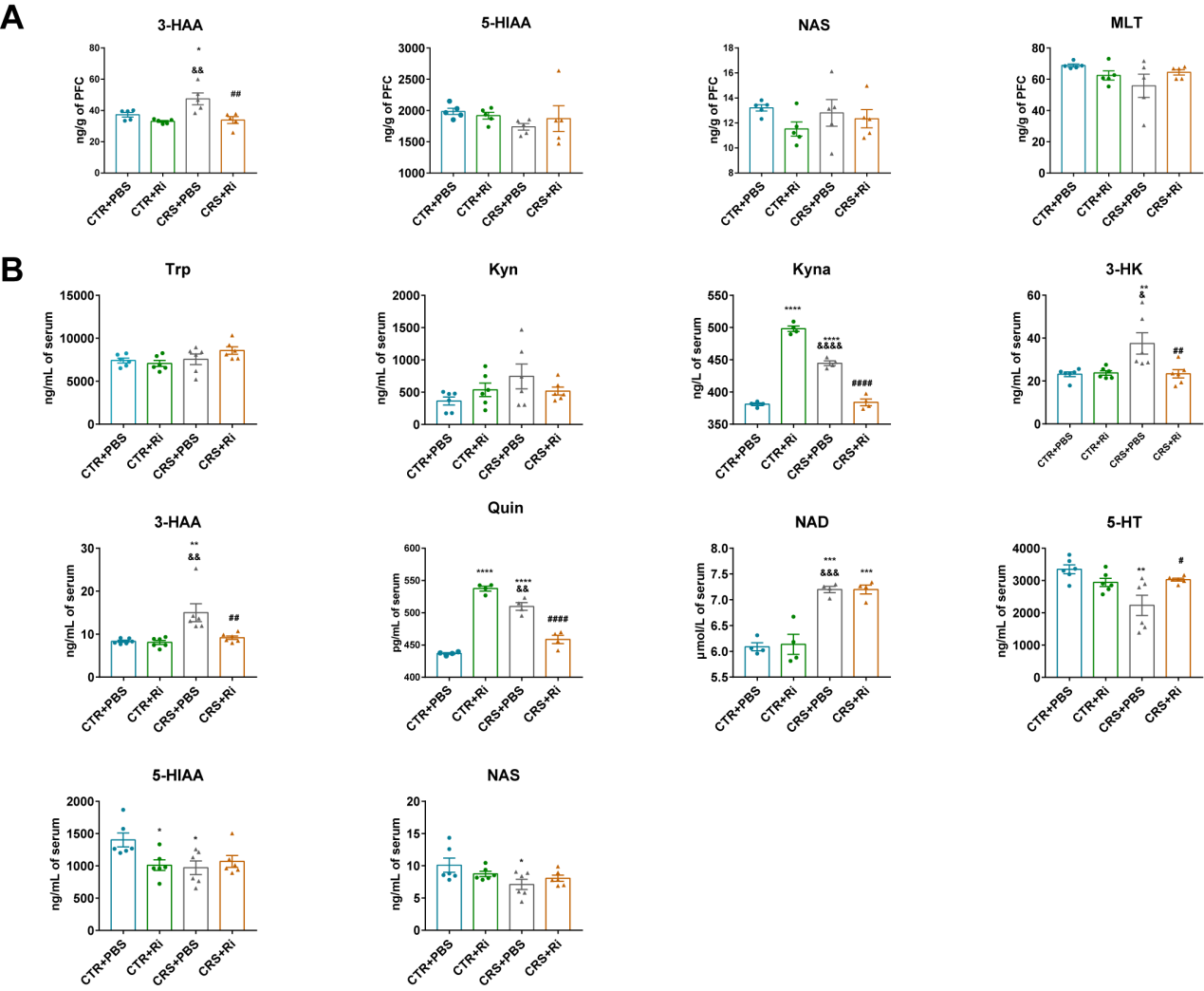


**Figure S4.** **Concentrations of neurotransmitters along Trp-derived metabolic pathways in *Ri*. intervened mice. (A)** Other downstream products of KP and Trp-5-HT branches in PFC. **(B)** Concentrations of neurotransmitters from Trp-Kyn and Trp-5-HT metabolic pathway in mouse serum. Data were represented as mean±SEM. ^*^p<0.05, ^**^p<0.01, ^***^p<0.001, ^****^p<0.0001 versus CTR+PBS group; ^&^p<0.05, ^&&^p<0.01, ^&&&^p<0.001, ^&&&&^p<0.0001 versus CTR+Ri group; ^#^p<0.05, ^##^p<0.01, ^####^p<0.0001 versus CRS+PBS group. All data here were analyzed by two-way ANOVA, Tukey’s multiple comparison test was applied for post hoc test.


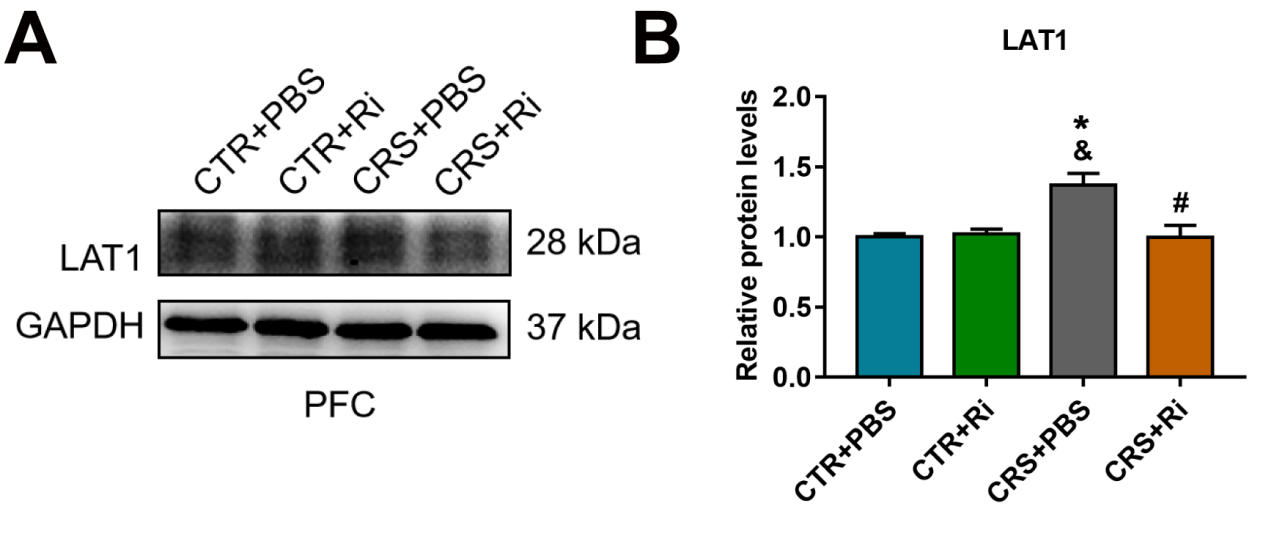


**Figure S5.** **Expression of large neutral amino acid transporter 1 (LAT1) in PFC and mucus changes in colon of the each treatment group.**

(A) LAT1 expression in PFC of *Ri*. gavage mice. (B) Statistical graph of LAT1.

Data were represented as mean±SEM. ^*^p<0.05, versus the CTR+PBS group; ^#^p<0.05 versus the CRS+PBS group. Significant differences were determined via two-way ANOVA, Tukey’s multiple comparison test was applied for post hoc test.

**Table S1**. Demographic characteristics of depressive and healthy control participants.

| Characteristic | HC (n = 10) | DEP t (n = 25) | *p* value |
| --- | --- | --- | --- |
| *Adolescent characteristics* | | | |
| Age (years) | 14.80±0.42 | 13.76 ±1.23 | *p*=0.014* |
| Body weight (kg) | 53.08±2.63 | 57.10±2.55 | *p*=0.365 |
| BMI | 21.02±1.03 | 21.42±0.94 | *p*=0.811 |
| *Parental characteristics* | | | |
| *Marital status (n (%))* | | | |
| Married | 10 (100) | 18 (72) | *p*=0.084 |
| Separated or divorced or widowed | 0 (0) | 7 (28) |  |
| *Paternal education level (n (%))* | | | |
| ≤junior high school | 5 (50) | 20 (80) | *p*=0.107 |
| ＞junior high school | 5 (50) | 5 (20) |  |
| *Paternal employment status (n (%))* | | | |
| Employed | 8 (80) | 15 (60) | *p*=0.434 |
| Unemployed | 2 (20) | 10 (40) |  |
| *Maternal education level (n (%))* | | | |
| ≤junior high school | 7 (70) | 18 (72) | *p*=1.000 |
| ＞junior high school | 3 (30) | 7 (28) |  |
| *Maternal employment status (n (%))* | | | |
| Employed | 9 (90) | 16 (64) | *p*=0.218 |
| Unemployed | 1 (10) | 9 (36) |  |
| *Gestational characteristics* | | | |
| Parity (n (%)) | | | |
| 1 | 10 (100) | 21 (84) | *p*=1.000 |
| ≥2 | 0 (0) | 3 (16) |  |
| Pregnant age | 24.60±2.76 | 25.96±4.57 | *p*=0.388 |
| Emotional state of pregnancy (n (%)) | | | |
| Fine | 10 (100) | 23 (92) | *p*=1.000 |
| Depressive | 0 (0) | 2 (8) |  |

Data were shown as mean±SEM. ^*^p＜0.05 versus the HC group. Except for age, body weight and BMI differences between HC and DEP t groups tested by Student’s *t*-test, other features were analyzed by chi-square test or Fisher’s exact test.

DEP t: Depressive adolescents total, including the DEP group and DEP sertraline-treated group.

**Table S2.** Specific primers sequences.

| Gene Name | Primer/Probe Sequence | |
| --- | --- | --- |
|  | Forward | Reverse |
| Lgr5 | GGACCAGATGCGATACCGC | CAGAGGCGATGTAGGAGACTG |
| Bmi1 | AAATCCCCACTTAATGTGTGTCC | CTTGCTGGTCTCCAAGTAACG |
| Msi1 | TAAAGTGCTGGCGCAATCG | TCTTCGTCCGAGTGACCATCT |
| Hopx | CATCCTTAGTCAGACGCGCA | AGGCAAGCCTTCTGACCGC |
| Muc1 | TACCCTACCTACCACACTCACG | CTGCTACTGCCATTACCTGC |
| Muc3 | CTTCCAGCCTTCCCTAAACC | TCCACAGATCCATGCAAAAC |
| Muc13 | CAACTCAGCCTTCTGGTGGT | TACAGGGGTTAGGGTTGCAG |
| Muc20 | CAACATCTGCATCCACTGCT | GTCAGCCGTACAAGGAGGAA |
| Muc5ac | ATCTTTCAGGACCCCTGCTC | ATGGACCACTGGCGTTAGTC |
| GAPDH | AGGTCGGTGTGAACGGATTTG | TGTAGACCATGTAGTTGAGGTCA |
| *Roseburia intestinalis* | CGGATTTGCAGTGGCAAGTT | TGATTGCAGACGCCAATGTC |
| Universal bacteria | ACTCCTACGGGAGGCAGCAGT | ATTACCGCGGCTGCTGGC |
| Roseburia cluster | Rint623 TTCCAATGCAGTACCGGG | |
